# Controls of plant diversity and composition on a desert archipelago

**DOI:** 10.7717/peerj.7286

**Published:** 2019-07-09

**Authors:** Benjamin T. Wilder, Richard S. Felger, Exequiel Ezcurra

**Affiliations:** 1 Desert Laboratory on Tumamoc Hill, University of Arizona, Tucson, AZ, USA; 2 University of Arizona Herbarium, University of Arizona, Tucson, AZ, USA; 3 Department of Botany and Plant Sciences, University of California, Riverside, Riverside, CA, USA

**Keywords:** Community composition, Cultural dispersal, Land-sea connections, Islands, Island biogeography, Species diversity

## Abstract

**Aim:**

With the most robust floristic data set for any arid archipelago, we use statistical modeling to determine the underlying controls of plant diversity and species composition.

**Location:**

The study was undertaken in the Midriff Islands of the Gulf of California, Mexico.

**Methods:**

Using the area–diversity relationship we estimate the power coefficient *z* with generalized linear models (GLM). We tested eight predictors (area, human presence, habitat diversity, topography, distance to mainland, island type, precipitation, and seabird dynamics) using a step-wise process on the same GLM procedure. Plant species composition was assessed by conducting a non-standardized principal component analysis on a presence-absence matrix of the 476 (plant species) × 14 (islands). Finally, families were tested for over or under representation with a *X*^2^ analysis subjected to a Bonferroni correction.

**Results:**

The classic species-area model explained 85% of the variance in island plant diversity and yielded a slope (*z*) of 0.303 (±0.01). When the effect of area is removed, four additional factors were shown to account for observed variation; habitat diversity (34%), seabird dynamics (23%), island type (21%), topography (14%). Human presence and distance to mainland were not predictors of species richness. Species composition varies significantly with island area; small islands have a particular flora where certain families are overrepresented, such as Cactaceae, while the flora of larger islands is strongly dependent on the continental source.

**Main conclusions:**

The factors that control diversity levels are expressions of geology, landscape heterogeneity, and land-sea connections. Species assemblages in small islands are governed by copious marine nutrients in the form of guano that depress species diversity. Distance to mainland and human presence hold no predictive power on diversity. The results show these islands to be isolated arid ecosystems with functioning ecological networks.

## Introduction

Islands have figured prominently in many of the greatest scientific advances in what Darwin termed the mystery of mysteries, the appearance of life in geologically recent environments (Darwin, 1860). The web of ecological complexity is simplified on island systems. Variables are reduced and responses and adaptations to causal factors are evident, allowing insights that are blurred elsewhere (Wallace, 1880). Driven by similar curiosities and a desire to harness the power of islands as model systems, Robert MacArthur and Edward O. Wilson changed the landscape of ecological thinking with their book *The Theory of Island Biogeography* (MacArthur & Wilson, 1967). They proposed a model to explain the factors that drive species richness in isolated natural communities. Their theory explaining the dynamics of species immigration, extinction, survival, and evolution in these enclosed microcosms promised a new approach for better understanding and managing the natural world. A scientific revolution was underway.

Possibly the most important aspect of the theory of island biogeography is the mathematical formalization of a simple species-area relationship as a fundamental paradigm in biogeography. Today, this platform allows continued insights and integration between a multitude of disciplines that makes synthetic biogeography the new standard (Warren et al., 2015; Santos, Field & Ricklefs, 2016; Patiño et al., 2017). Here, inspired by the potential of diverse data sets within the framework of island biogeography theory we aim to better understand the factors that account for the given diversity across islands and what can create a set of distinct island worlds even within an archipelago.

The islands of the Gulf of California, Mexico are a powerful test bed for MacArthur and Wilson’s theory, arguably as important as the Galápagos (Case & Cody, 1983a). The islands occur in the arid Sonoran Desert and exist along a wide gradient of island size and geologic origin. Nearly every way to be an island (landbridge, oceanic, recent, old, large small, etc.) is represented in the Gulf of California. Likewise, the marine environment contains some of the highest primary productivity values in the world (Douglas et al., 2007), presenting a striking contrast of arid lands in the midst of marine abundance and diversity.

Utilizing a robust data set from early expeditions that surveyed the biological richness of the gulf islands across taxa (Townsend, 1916; Johnston, 1924; Gentry, 1949; Lindsay, 1955; Felger, 1966; Lindsay, 1966), Martin Cody and Ted Case organized in 1977 a symposium on the islands of the Gulf at the University of California, Los Angeles. From the papers presented, they edited a seminal book titled *Island Biogeography in the Sea of Cortez* (Case & Cody, 1983a). In that volume they underscored the great biological value of the gulf islands as a model system. They also acknowledged that, apart from the equilibrium between immigration and extinction as the major factor describing insular biodiversity, other processes such as interspecific interactions or historical legacies could be equally or more important. In particular, they proposed two areas for improvement of the theory (Case & Cody, 1983b).

Firstly, Case & Cody (1983b), as others have since (Warren et al., 2015), questioned the random colonization hypothesis; namely, the view implicit in the equilibrium model that islands are simple, small pieces of the mainland providing similar habitats and resources over a circumscribed area. Small islands, in particular, have their own characteristic environments and interactions with the surrounding ocean waters often deviating from the expected positive species-area relationship (Whitehead & Jones, 1969; Barrett, Wait & Anderson, 2003).

Because the species-area model is strongly based on a species accumulation function, it implicitly treats all species from the pool in the continental source as having the same probability of colonizing an island of any size. MacArthur & Wilson (1967: 56) put forward a random colonization hypothesis stating that, “in fact, since all species are equally probable, the *S* species on the first island are a random sample of the *P* available [from the source].” As discussed by Case & Cody (1983b) and more recently Santos, Field & Ricklefs (2016) and Patiño et al. (2017), this may be a somewhat unrealistic assumption, especially in small islands that have a very high perimeter-to-area ratio and are highly influenced by ocean dynamics (Polis et al., 1997; Anderson & Wait, 2001). Clearly, most continental species cannot thrive in the extreme maritime environment of small oceanic islands, and the process of colonization in these islands may be driven by other factors differing significantly from the assumption of random colonization in the equilibrium model.

Case and Cody’s second concern derives from the first one. Because the equilibrium model of island biogeography assumes that island colonization is basically a random species accumulation process, it pays little attention to the problem of species composition on the different islands. With great intellectual honesty, MacArthur & Wilson (1967: 56) conceded that “knowledge on the *number* [sic] of species on islands of the same area […] can provide an idea of the degree to which equilibrium has been approached. So far extra knowledge of the *names* [sic] of the species has been wasted.” If the environment of small islands is fundamentally different from that of the mainland, then species colonizing small islands will form a distinct assemblage that can be quite different from those found on the mainland. Classifying islands simply by the number of species found there could hide an important part of the evolution and dynamics shaping island biotas. All insular species are not equal, as implied by the immigration and extinction curves used in the equilibrium model. Looking in some detail at the composition of the biota on different islands may provide an interesting means of evaluating the true nature of the species accumulation process at different spatial scales.

This paper follows from these two observations of Case and Cody and recent reviews upon the 50th anniversary of MacArthur and Wilson’s publication for next steps in better understanding the origin of island diversity. Based on over a century of comprehensive plant collecting in the Gulf of California Midriff Islands (Felger, Wilder & Romero-Morales, 2012; Wilder, 2014; Appendix S1), we revisit the explanatory power of the theory of island biogeography to determine the factors driving species diversity on the islands. We focus first on species numbers, using regression methods to understand the influence of island area and of other factors such as pre-historic human presence, habitat diversity, topography, distance to the mainland, island type (oceanic vs. landbridge), precipitation, and seabird dynamics on total species richness. Second, we examine the floristic composition of the islands using community ecology methods to understand the influence of different factors on the species assemblages present on each island. We focus our attention on plants, the majority of which are readily dispersed, fairly persistent (Case & Cody, 1987), and responsive to short- and long-term environmental conditions.

## Materials and Methods

### Study area: the Midriff Islands

**Figure 1:**
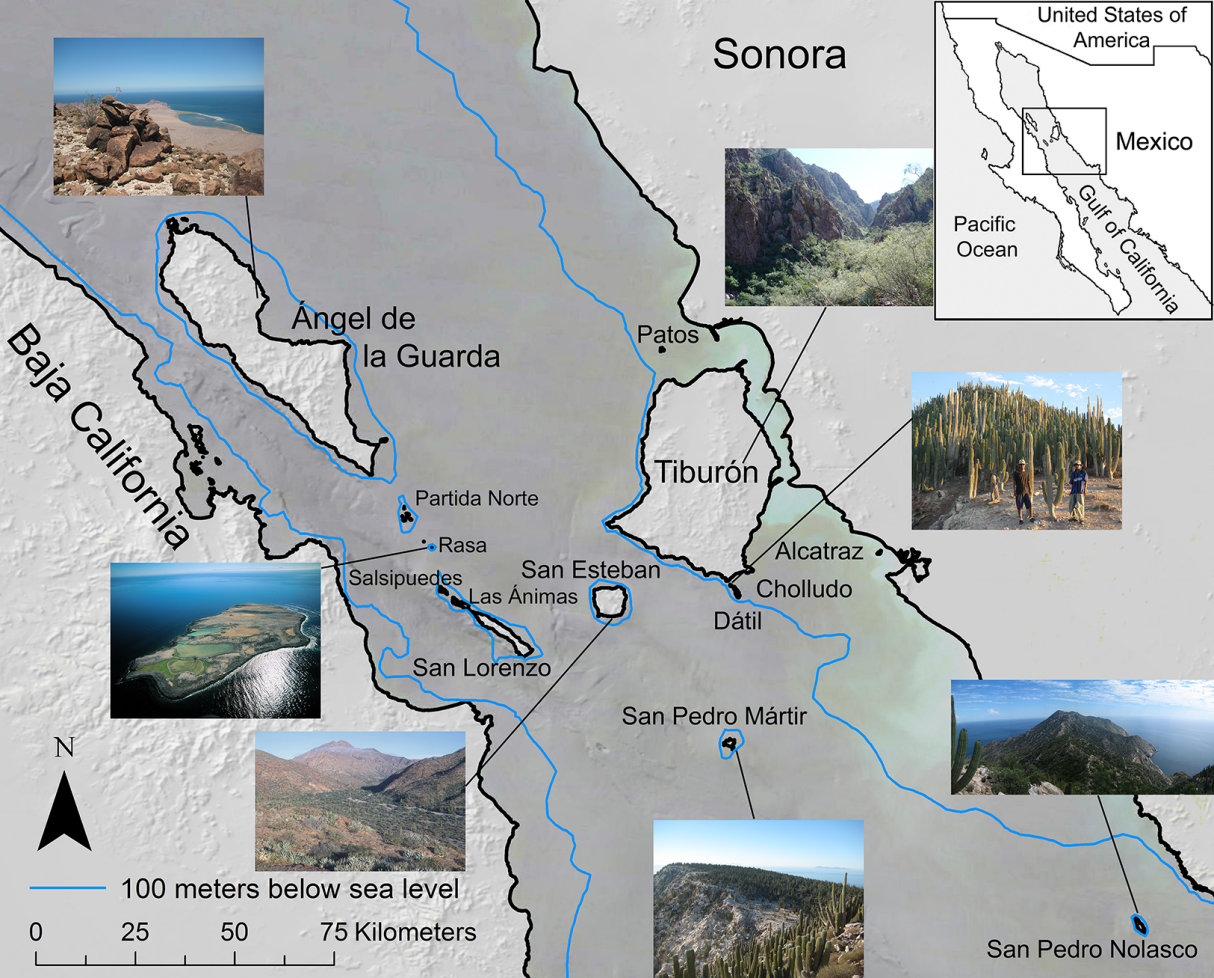
Midriff Islands of the Gulf of California, Mexico. The 100 m bathometric line indicates approximate coastline at the last glacial maximum. Photos by Wilder except Isla San Esteban by Felger and Isla Rasa by Fulvio Eccardi. Gulf of California DEM and hill shade GIS layers provided by Scott Bennett and satellite imagery courtesy of NASA© 2010. Inset regional map by Cathy Moser–Marlett.

The Midriff Islands in the Gulf of California, Mexico (Fig. 1) are an isolated, arid, and long-inhabited archipelago. This set of 14 islands that span the Gulf of California, or Sea of Cortés, from the Baja California peninsula to the Mexican mainland occur within the Central Gulf Coast subdivision of the Sonoran Desert (Shreve, 1951). The islands range in size from 0.2 km^2^ (Isla Cholludo) upward to 1,223 km^2^ (Isla Tiburón, the largest island in Mexico), reaching highest elevations of 1,316 m on Isla Ángel de la Guarda and 885 m on Tiburón (Felger, Wilder & Romero-Morales, 2012). They have been inhabited or visited by the sea faring and hunter-gatherer Comcaac (Seri people), or their ancestors or predecessors, for millennia (Felger & Moser, 1985; Bowen, 2000). Geologically, the Gulf of California dates to ca. six Ma (Ledesma-Vázquez & Carreño, 2010) and is one of the world’s most productive oceans (Douglas et al., 2007). Today the islands are a UNESCO World Heritage site, a Mexican natural protected area (DOF, Diario Oficial de la Federación, 2000), and since the 1950s uninhabited. For a comprehensive review of the Gulf of California islands, see Case, Cody & Ezcurra (2002). Collections were made under Mexican federal collecting permit NOM-126-SEMARNAT-2000 issued to Dr. Ezcurra.

### Species-area model

Like many other scaling phenomena in biology, the relationship is usually described by a power function of the form *S* = *kA^z^*, where *S* is the number of species on an island, *A* is island area, *z* is the power exponent, and *k* is a scale coefficient. In practice, the species-area curve is an accumulation function similar to those used to evaluate the completeness of herbarium collections (Soberón & Llorente, 1993) and is based on Preston’s canonical model (Preston, 1962). The theory on biological scaling has dramatically expanded since 1967 (Brown & West, 2000), but the basic species-area model, based on MacArthur and Wilson’s “equilibrium theory” of island biogeography is still one of the most robust models of ecological theory (see Gould, 1979 for a review).

To estimate the power coefficient *z* of the area–diversity relationship for the Midriff Islands, we used generalized linear models (GLM), a novel approach in species-area relationships. Because our dependent variable, the number of species on each island, is a count variable that is expected to have a Poisson error distribution, we used Poisson regression (also known as log-linear regression), in which the fit of the model to the data is measured as a log-likelihood function with a χ^2^ distribution of the error (McCullagh & Nelder, 1989).

The derivation of our model is as follows (see Appendix S2 for an alternative derivation): The species-area model for islands *S* = *kA^z^* can be rewritten in log-log form: log *S* = log *k* + *z* log *A*. This is a linear model where log *k*, the intercept, can be also expressed as a constant parameter *b* = log *k*. Raising the exponent of both sides we get: *S* = *e*^(*b*+*z* log *A*)^, a log-linear function in which the count variable *S* is expressed as the exponent of the linear function *b* + *z* log *A*. The GLM algorithm identified the values of *b* and *z* that minimize the error of the fit. The error in turn is calculated as a log-likelihood deviance function: ${\rm{\varepsilon }} = 2\mathop \sum \nolimits^ \,{S_i}\,{\rm{log}}\left( {{S_i}/{{\hat S}_i}} \right)$ that is numerically convergent to the more familiar chi-squared statistic: ${X^2} = \,\mathop \sum \nolimits^{} \,\left[ {{{\left( {{S_i} - {{\hat S}_i}} \right)}^2}{\hskip-4pt}/{\hskip-0.2pt}{{\hat S}_i}} \right]$ (in both equations, *S_i_* is the number of species observed in island *i*, and ${\hat S_i}$ is the number of species predicted by the model for island *i*). In short, we fitted a Poisson GLM to the species richness of the islands, using the logarithm of the area as the predictor. The slope and standard error of the fitted function gave us the value of *z* and its error.

Because in a Poisson model the variance is equal to the mean, each individual Chi-squared error term $\left[ {\left({{S_i}-{{\hat S}_i}} \right)/\sqrt {{{\hat S}_i}} } \right]$ is distributed as a normal-distribution deviate $\left[ {z = \left({x-{\rm{\mu }}} \right)/{\rm{\sigma }}} \right]$ that can be tested against the critical values of the Normal curve (i.e., errors are Pearson residuals, see Duffy, 1990). Using this property, we were able to identify islands that had significantly more or significantly fewer species than predicted by the equilibrium model of island biogeography.

### Predictors of species richness

After confirming that some islands depart significantly from the species-area model, we tested additional predictors of species richness for the archipelago (Table 1), using a step-wise process on the same GLM procedure. The predictors were: (a) presence of archeological artifacts (yes or none found) as a surrogate for prehistoric human presence, (b) habitat diversity (or number of distinct habitats; a number between 1 and 14), (c) topography (subtle, moderate, or rugged relief), (d) distance to the nearest mainland, and distance to the nearest island (in km), (e) island type (oceanic or landbridge), (f) seabird dynamics (presence/absence of nesting seabirds), and (g) precipitation. Some of our predictors were continuous variables while others were categorical.

**Table 1:** Factors of diversity.

Island	^1^Number of plant species	^2^Area (km^2^)	^3^Habitat diversity	^4^Topography	^5^Distance (km)	^6^Island type	^7^Precipitation (mm)	^8^Seabird rookery	^9^Archaeological evidence
Tiburón	^1a^349	1,223	14	Rugged	^5a^1.70	^6a^Land bridge	119	^8a^No	Yes
Ángel de la Guarda	^1b^217	936	10	Rugged	^5b^12.12	^6b^Oceanic	65	^8b^No	Yes
San Esteban	^1c^114	41	9	Rugged	^5c^11.64, 16.85, 34.5	^6c^Oceanic	114	^8c^No	Yes
San Lorenzo (San Lorenzo Sur)	^1d^85	33	4	Moderate	^5d^16.36	^6d^Oceanic	91	^8d^No	Yes
Las Ánimas (San Lorenzo Norte)	^1e^45	4.26	1	Moderate	^5e^16.36	^6e^Oceanic	88	^8e^Yes	Yes
Nolasco	^1f^58	3.45	5	Rugged	^5f^14.61	^6f^Oceanic	186	^8f^No	None known
Mártir	^1g^29	2.67	3	Moderate	^5g^39.09, 50, 50	^6g^Oceanic	111	^8g^Yes	None known
Alcatraz	^1h^54	1.44	4	Moderate	^5h^2.01	^6h^Landbridge	140	^8h^Yes	Yes
Partida Norte	^1i^18	1.36	2	Moderate	^5i^8.30, 12.18, 17.88	^6i^Oceanic	87	^8i^Yes	Yes
Dátil	^1j^101	1.25	4	Rugged	^5j^1.94	^6j^Landbridge	121	^8j^No	Yes
Salsipuedes	^1k^18	1.16	1	Subtle	^5k^1.52, 19.21	^6k^Oceanic	87	^8k^Yes	Yes
Rasa	^1l^14	0.68	1	Subtle	^5l^20.79	^6l^Oceanic	88	^8l^Yes	None known
Patos	^1m^14	0.45	2	Subtle	^5m^7.45, 8.82	^6m^Landbridge	126	^8m^Yes	None known
Cholludo	^1n^31	0.2	3	Moderate	^5n^1.09	^6n^Landbridge	121	^8n^Yes	None known

**Notes:**

The 14 islands considered, number of plant species and categorization or metrics for the eight factors of diversity tested.

Islands are listed in order of decreasing area.

1 Flora totals as seen in Appendix S1 are from: ^1a^Felger, Wilder & Romero-Morales (2012) and Wilder (2014); ^1b^Moran (1983), Rebman (2010), and Wilder (2014); ^1c^Felger, Wilder & Romero-Morales (2012); ^1d^Rebman, León de la Luz & Moran (2002) and Wilder (2014); ^1e^Rebman, León de la Luz & Moran (2002), and Wilder (2014); ^1f^Felger, Wilder & Gallo-Reynoso (2011), Felger, Wilder & Romero-Morales (2012); ^1g^Wilder & Felger (2010), Felger, Wilder & Romero-Morales (2012), and Wilder (2014); ^1h^Felger, Wilder & Romero-Morales (2012); ^1i^Rebman, León de la Luz & Moran (2002), and Wilder (2014); ^1j^Felger, Wilder & Romero-Morales (2012); ^1k^Rebman, León de la Luz & Moran (2002), and Wilder (2014); ^1l^Velarde et al. (2014); ^1m^Felger, Wilder & Romero-Morales (2012); ^1n^Felger, Wilder & Romero-Morales (2012), and Wilder (2014).

2 Island size from Murphy et al. (2002).

3 Habitat classes that affect the 14 vegetation types found on the Midriff Islands (Felger, Wilder & Romero-Morales, 2012): ridges, arroyos, canyons, permanent springs and ephemeral tinajas, coastal bajada, foothill bajada, peaks above 500 m, coastal area and salt flats, flats, north-facing slopes, esteros, sea cliffs, valleys, dunes.

4 Topography classes are based on the authors understanding of the relief and elevation (Murphy et al., 2002; Felger, Wilder & Romero-Morales, 2012) of each island; they capture the complexity, or lack there of, of insular terrains.

5 Distances (Murphy et al., 2002) to nearest large land body: ^5a^Sonora; ^5b^Peninsula ^5c^Tiburón, San Lorenzo, Peninsula; ^5d^Peninsula; ^5e^Peninsula; ^5f^Sonora; ^5g^Tiburón, Sonora, Peninsula; ^5h^Sonora; ^5i^Rasa, Ángel de la Guarda, Peninsula; ^5j^Tiburón; ^5k^Las Ánimas, Peninsula; ^5l^Peninsula; ^5m^Tiburón, Sonora; ^5n^Tiburón.

6 Classification of landbridge or oceanic based on geologic origin information from Carreño & Helenes (2002; except where stated otherwise), and age since last isolation as indicated: ^6a^Faulting, uplift, and erosion, ca. 6,000 ya (Wilcox, 1978; Lambeck & Chappell, 2001; Davis, 2006; Felger, Wilder & Romero-Morales, 2012); ^6b^Block Faulting, 3.3–2 Ma (Aragón-Arreola & Martín-Barajas, 2007; Nagy & Stock, 2000; Stock, 2000); ^6c^Volcanic (Desonie, 1992), 2.9–2.5 Ma (Desonie, 1992); ^6d^Block Faulting, 3.3–2 Ma (Aragón-Arreola & Martín-Barajas, 2007; Nagy & Stock, 2000; Stock, 2000); ^6e^Block Faulting, 3.3–2 Ma (Aragón-Arreola & Martín-Barajas, 2007; Nagy & Stock, 2000; Stock, 2000); ^6f^Faulting, 3–2 Ma (Felger, Wilder & Gallo-Reynoso, 2011); ^6g^Volcanic, no age data is available but is presumably a similar age as San Esteban; ^6h^Faulting, uplift, and erosion, ca. 6,000 ya (Wilcox, 1978; Lambeck & Chappell, 2001; Davis, 2006; Felger, Wilder & Romero-Morales, 2012); ^6i^Volcanic, no age data is available but is presumably similar to adjacent Salsipuedes; ^6j^Faulting, uplift, and erosion, ca. 6,000 ya (Wilcox, 1978; Lambeck & Chappell, 2001; Davis, 2006; Felger, Wilder & Romero-Morales, 2012); ^6k^Block Faulting, 3.3–2 Ma (Aragón-Arreola & Martín-Barajas, 2007; Nagy & Stock, 2000; Stock, 2000); ^6l^Volcanic, 10,000 ya (Velarde et al., 2014); ^6m^Faulting, uplift, and erosion, ca. 6,000 ya (Wilcox, 1978; Lambeck & Chappell, 2001; Davis, 2006; Felger, Wilder & Romero-Morales, 2012); ^6n^Faulting, uplift, and erosion, ca. 6,000 ya (Wilcox, 1978; Lambeck & Chappell, 2001; Davis, 2006; Felger, Wilder & Romero-Morales, 2012).

7 Precipitation values are based on an extrapolation from long-term precipitation data from six coastal meteorological stations maintained by the Mexican government. See methods section for more detail.

8 Seabird usage based on cumulative knowledge as identified: ^8a^Cody & Velarde (2002); ^8b^Cody & Velarde (2002); ^8c^Cody & Velarde (2002); ^8d^considered to have a large colony of seabirds (Sanchez-Piñero & Polis, 2000) where nesting is confined to the northern third of island, primarily pelicans (Dan Anderson, August 16, 2014, personal communication); ^8e^nesting is island wide, primarily pelicans (Dan Anderson, August 16, 2014, personal communication); ^8f^not considered a seabird island (Felger, Wilder & Gallo-Reynoso, 2011; Dan Anderson, August 16, 2014, personal communication); ^8g^a significant seabird island with eight breeding seabirds species, especially blue footed and brown boobies (Tershy & Breese, 1997); ^8h^southwestern portion of island supports 11 breeding species, especially Double-crested Cormorants (Duberstein et al., 2005); ^8i^significant seabird island (Sanchez-Piñero & Polis, 2000) with at least five breeding species, especially Craveri’s Murrelet (Velarde et al., 2005), Least Storm-Petrel (Velarde, 2000), occasionally brown pelicans (D. Anderson and T. Bowen, August 16, 2014, personal communication), and the largest population of fishing bats (*Myotis vivesi*) in the Gulf of California (Maya, 1968; Velarde et al., 2005); ^8j^not considered a seabird island (Dan Anderson, August 16, 2014, personal communication); ^8k^nesting over the total island, but quite spotty (D. Anderson, August 16, 2014, personal communication), we follow (Sanchez-Piñero & Polis, 2000) in designating this a seabird island; ^8l^a significant seabird island (Sanchez-Piñero & Polis, 2000) especially Heermann’s Gull, Elegant Terns, and Royal Terns (Velarde, 1989; Cody & Velarde, 2002; Velarde et al., 2014); ^8m^Nesting over the total island, sporadic (D. Anderson, August 16, 2014, personal communication), and perhaps not recovered from vegetation removal for guano harvesting in 1946 (Felger, Wilder & Romero-Morales, 2012; Dan Anderson, August 16, 2014, personal communication); ^8n^Total island, but spotty within cardón forest (D. Anderson and Enriqueta Velarde, August 16, 2014, personal communication).

9 Presence of archaeological remains is based on Bowen (2009).

Data for the predictors are as follows (full sources can be seen in Table 1): (a) The presence of humans on the islands was incorporated into our biogeographic analysis via archaeological investigations undertaken on all islands in question (Bowen, 2009). (b) While area and habitat diversity are correlated (Sfenthourakis & Triantis, 2009), and hence not truly independent, they may jointly have a significant additive effect (Hortal et al., 2009). For example, small islands can have a high degree of topographic complexity despite their size. In Sonoran Desert ecosystems, topographical diversity is strongly related to habitat and plant diversity (Búrquez et al., 1999). Following Hortal et al. (2009) we defined habitat diversity as the number of physiognomic vegetation types on each island as described by Felger, Wilder & Romero-Morales (2012). (c) Topography was taken into consideration by assigning each island to one of the three categories of relief based on highest elevation and our experience on the islands. (d) Isolation by distance was taken into account via an island’s proximity to the nearest mainland (Baja California peninsula or Sonora) and in several cases neighboring large island(s) that may serve as sources for immigration. (e) Geologic history of the island was incorporated via island type: landbridge or oceanic. (f) Given the preponderance and importance of nesting seabirds in the Gulf of California, especially on small islands (Velarde et al., 2005), we classified each island as a seabird island if there is annual presence of over ca. 5,000 breeding individuals and characteristic guano white-washed rocks and soil following Anderson & Polis (1999). (g) Because reliable and long-term climatological or meteorological data do not exist for the Midriff Islands, we used statistical extrapolations of long-term precipitation data from six coastal meteorological stations maintained by the Mexican government. Three stations were selected on the Gulf coast of Baja California (N–S: San Felipe, Bahía de los Ángeles, and Santa Rosalía, Ruiz Corral et al., 2006a, 2006b) and three from the Sonoran coast (N–S: Puerto Libertad, CONAGUA, 2010; Bahía de Kino, Ruiz Corral et al., 2005; and Guaymas, García et al., 1973). Precipitation for individual islands was calculated using linear interpolation between the nearest three stations to each island.

Island plant diversity was modeled using GLM in R (R Core Team, 2016). At each step of the regression analysis we tried all the variables that could be added, then selected the best fit using Akaike’s Information Criterion. We continued to add variables until no variable could significantly improve the fit.

### Species composition

To examine patterns in plant species composition across the 14 islands, a checklist of the flora of the Midriff Islands (Wilder, 2014; Appendix S1) was converted into a 476 (species) × 14 (islands) presence-absence matrix. Both species richness and species composition analyses include non-native species (only 12 non-native taxa occur in the flora). A non-standardized principal component analysis (PCA) was conducted on this matrix. Jackson’s (1993) broken-stick test was used to determine significant axes. To interpret the floristic variation captured by each axis in terms of external driving variables, we regressed the site scores of each axis against the same seven predictors (a–g) used for species richness. Five small islands were omitted from the distance-to-the-source analysis because the majority of their flora is formed by widespread coastal halophytes that obscure other patterns.

Using Noy-Meir’s (1973) divisive polythetic classification method, we partitioned the species along the first two significant PCA axes into floristic groups with minimum intragroup and maximum intergroup variation, numerically searching for partition thresholds that minimized intragroup variance. To visualize the distributional similarities of the floristic assemblages obtained, the centroids of these groups in the first two PCA axes were then clustered into a hierarchical dendrogram using the procedure *hclust* in R with average-linkage clustering and Euclidean distance (R Core Team, 2016).

Following Baselga (2010) we performed an evaluation of floristic nestedness on the island data to test whether small islands contain a flora that is distinct from that of the large islands, or if, alternatively, the small island flora is a nested subset of that in the large islands. We also tested whether the flora of small islands, if nested, is a random subset of the larger islands or a non-random collection of large island species. For this analysis, we used Monte–Carlo simulations randomly extracting two vectors with a probability of containing a mean of 19 species per simulated island, and comparing the Sorensen similarity among them. We did this 1,000 times, and the mean similarity and standard deviation of the simulated island pairs was compared to the true mean similarity among small islands in our data set.

Finally, to test whether certain families were overrepresented in some islands, we used a *X*^2^ analysis of the islands × families matrix with the number of species present in each family and each of the Midriff Islands. Probability values were subject to a Bonferroni correction to adjust for bias in multiple comparisons. Family classifications follow the Angiosperm Phylogeny Group IV system (Angiosperm Phylogeny Group, 2016) and recent work by Stevens (2019), reflecting current knowledge of evolutionary relationships.

## Results

### Predictors of species richness

**Figure 2:**
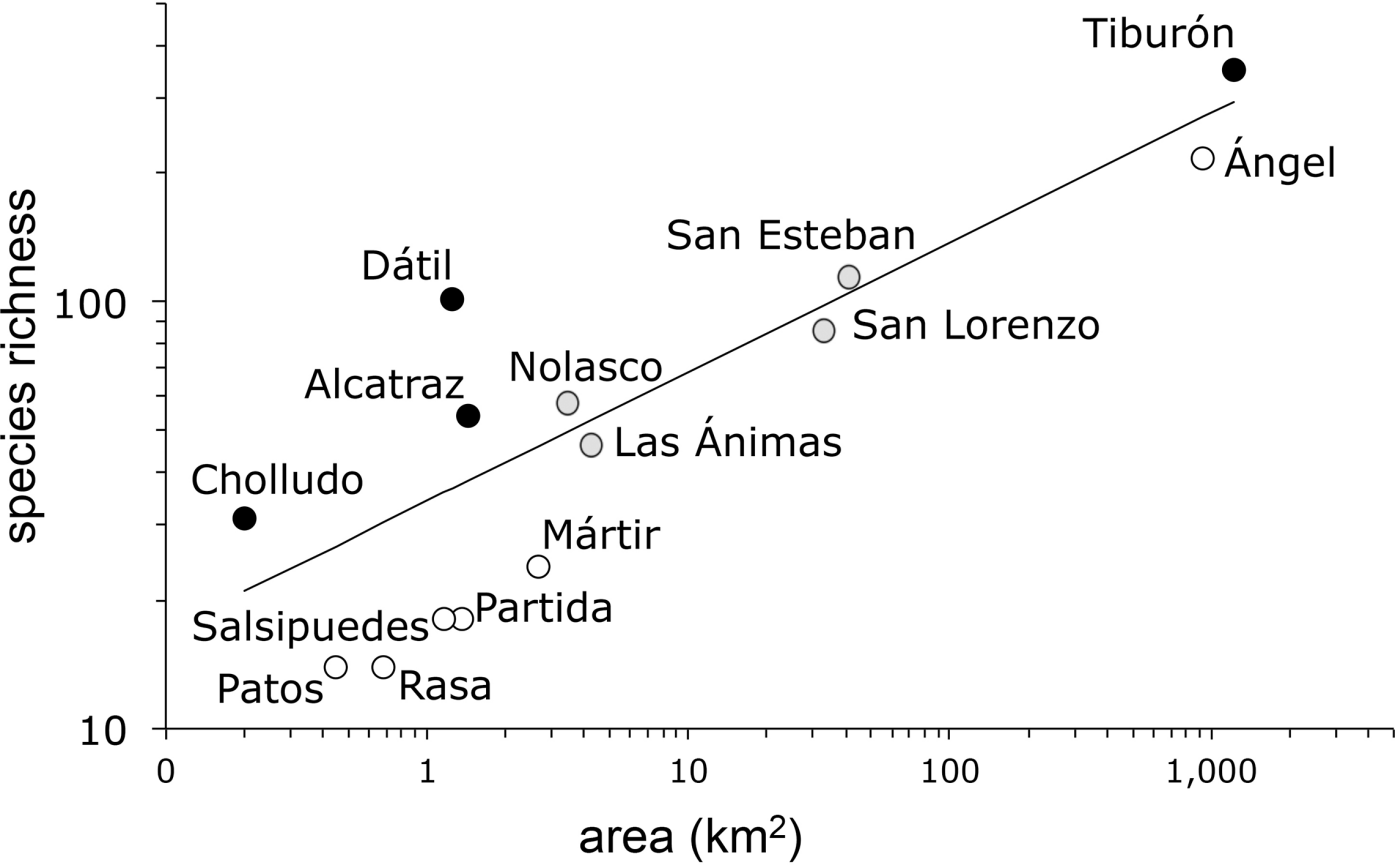
Plant species-area relationship for the Midriff Islands, Gulf of California, Mexico. Both axes are log transformed. The slope of the line (*z*) is 0.303 (s.e. ± 0.01) with an *r*^2^ = 0.85. Islands with significantly more species indicated by black circles, islands with significantly less species denoted by open circles, and islands with expected plant species diversity marked with gray circles.

The species-area model (Fig. 2) yielded a Poisson regression line with *r^2^* = 0.85 and a slope *z* = 0.303 (s.e. ± 0.01). As is frequently the case in island ecosystems, the value of the estimated species-area exponent (0.303) was significantly higher than 0.263, Preston’s canonical value expected for random species accumulation in terrestrial ecosystems (*t* = 4.0, d.f. 12, *P* = 0.0009). The analysis of the residuals identified four islands with more plant species than expected based solely on their area: Tiburón, Dátil, Alcatraz, and Cholludo, all part of the Tiburón landbridge archipelago. Six islands were shown to have significantly less species than expected: Ángel de la Guarda, San Pedro Mártir, Partida Norte, Patos, Salsipuedes, and Rasa. Apart from Ángel de la Guarda, each of these species-poor islands can be characterized as bird guano islands. Furthermore, all of them, except Patos, are true oceanic, not landbridge, in their geologic origin. Finally, four other islands, San Esteban, Las Ánimas, San Lorenzo, and Nolasco fell within the predicted range of the species area curve (Table 2).

**Table 2:** Pearson residual analysis for plant species-area relationship.

Island	Area (km^2^)	Number of species (*S*)	Expected *S*	Pearson residual	*P*
Tiburón	1,223	349	294	**3.25**	**0.0006**
Ángel de la Guarda	936	217	271	**−3.30**	**0.0005**
San Esteban	41	114	105	0.86	0.1951
San Lorenzo (San Lorenzo Sur)	33	85	99	−1.36	0.0866
Las Ánimas (San Lorenzo Norte)	4.26	45	53	−0.96	0.1692
Nolasco	3.45	58	50	1.18	0.1192
Mártir	2.67	29	46	**−3.24**	**0.0006**
Alcatraz	1.44	54	38	**2.57**	**0.0051**
Partida Norte	1.36	18	37	**−3.18**	**0.0007**
Dátil	1.25	101	37	**10.67**	**0.0000**
Salsipuedes	1.16	18	36	**−2.96**	**0.0015**
Rasa	0.68	14	30	**−2.97**	**0.0015**
Patos	0.45	14	27	**−2.47**	**0.0067**
Cholludo	0.2	31	21	**2.19**	**0.0142**

**Note:**

Pearson residual scores and probabilities for islands with significantly more or fewer species than expected by chance are shown in boldface.

After fitting the effect of island area on species richness, four additional predictors accounted for a significant amount of the residual variation (Table 3): (1) habitat diversity, 34%, (2) seabird rookery, 23% (3) island type (oceanic vs. landbridge), 21%, and (4) topography, 14%. Together, all these factors accounted for 92% of the observed variation in species richness, and the residual variation (8%) was not significant, matching the assumptions of a random Poisson distribution. Two other factors, distance to the source and human presence, had no significant effect in accounting for the observed variation.

**Table 3:** Analysis of variance.

Source of variation	χ^2^ deviance	d.f.	*P*	*r*^2^	*r*^2^ (removing effect of area)
Area	936.0	1	<0.0001	0.85	
Habitat diversity	57.8	1	<0.0001	0.05	0.34
Seabird rookery	38.1	1	<0.0001	0.03	0.23
Island type	34.5	1	<0.0001	0.03	0.21
Topography	24.1	2	<0.0001	0.02	0.14
Residuals	13.2	7	0.07	0.01	0.08
Total	1,103.6	13			

**Note:**

Results for the factors of diversity identified to significantly account for the variation from expected plant species diversity.

### Species composition

**Figure 3:**
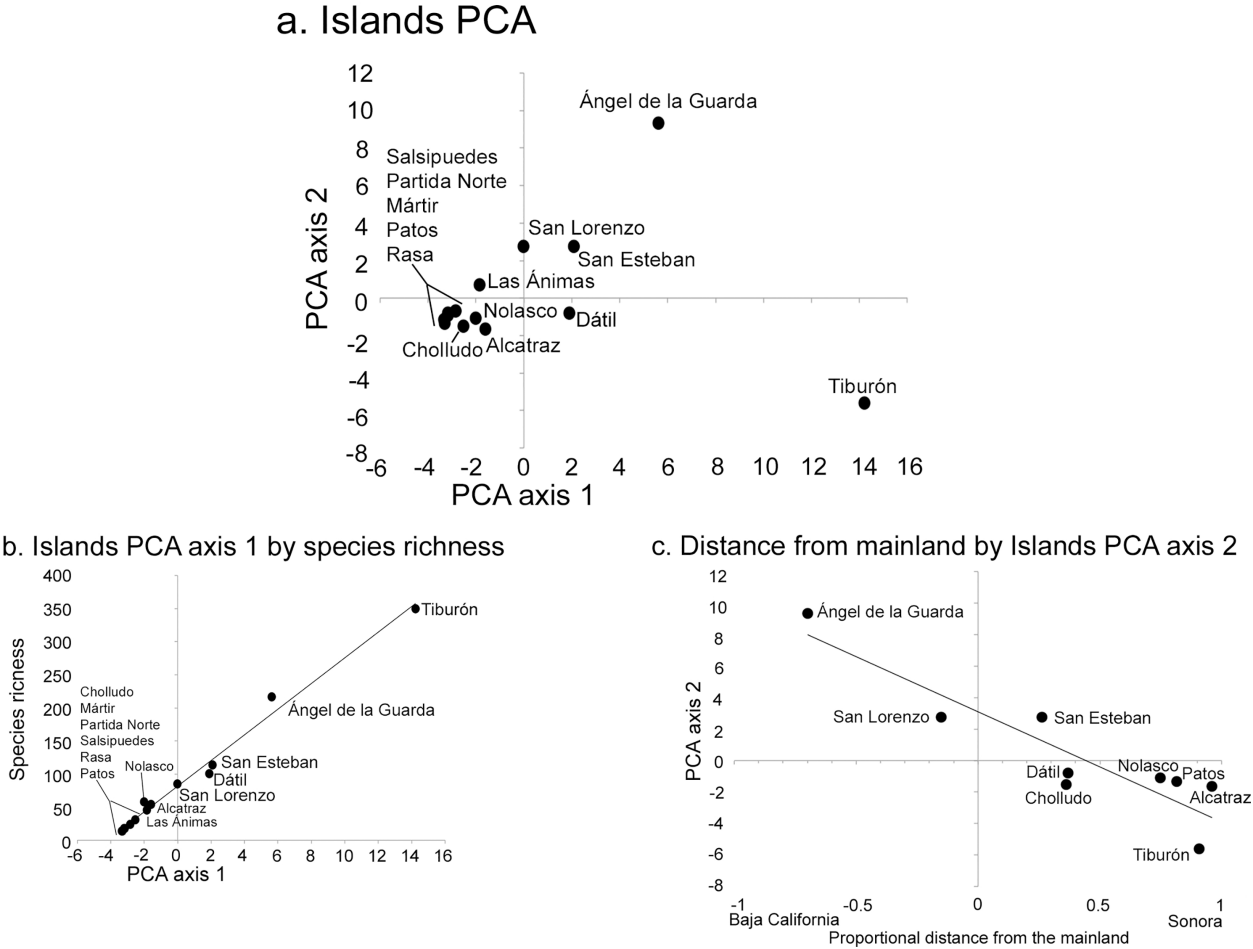
Midriff Island results of Principal Component Analysis (PCA). (A) PCA axes 1 and 2 show the 14 islands as subsets of the floristic matrix. (B) Correlation between PCA axis 1 and species richness (*r*^2^ = 0.99). (C) Correlation between PCA axis 2 and the proportional distance from the Baja California peninsula and Sonoran mainland (*r*^2^ = 0.74). Negative *x*-axis values reflect islands closer to Baja California, positive values are closer to Sonora.

**Figure 4:**
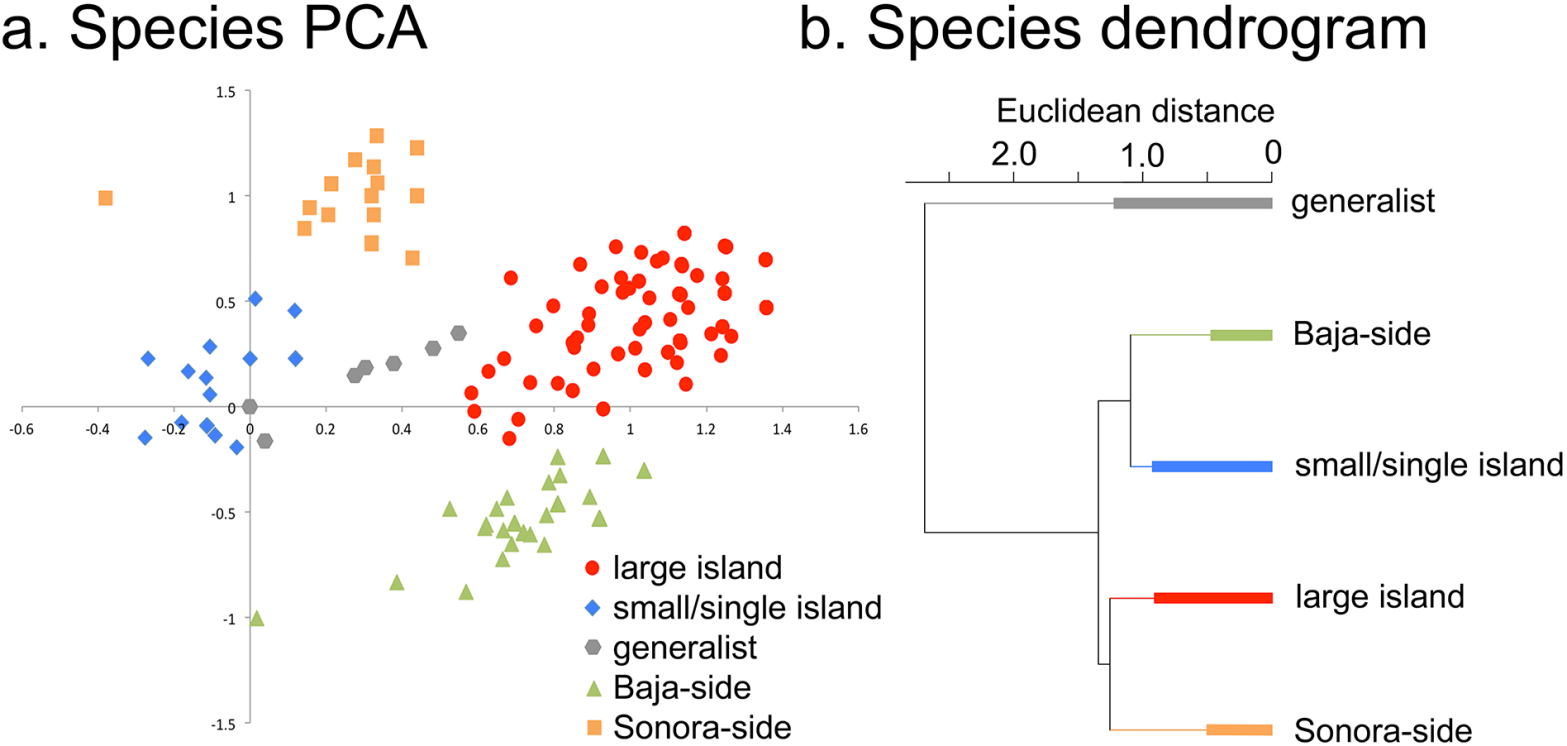
Individual species results of Principal Component Analysis (PCA). (A) Species PCA, the 476 plant taxa of the Midriff Islands distributed along PCA axes 1 and 2. Coding for the five species categories is based on the floristic checklist for the Midriff Islands (Wilder, 2014). (B) Species divisive dendrogram, relationship of the five species categories identified in (A). The bars in each branch of the dendrogram show the residual within-group variance.

The PCA analysis had two significant axes as determined by the broken-stick distribution model (Jackson, 1993). The first axis explained 41% of the overall floristic variation in the dataset and the second 19% (broken-stick expected values were 23% and 16%, respectively). The PCA simultaneously analyzes the relationship between islands, each with their component species (Fig. 3), or the distribution patterns of species among islands (Fig. 4). When islands are assessed, the first axis is strongly correlated with species richness (*r*^2^ = 0.99; Fig. 3B), while the second axis displays a floristic-composition gradient in the large islands from west (Baja California, Ángel de la Guarda) to east (Sonoran mainland, Tiburón; *r*^2^ = 0.74; Fig. 3C); the floristic composition of the small islands was independent of distance to the source. This second PCA axis was also correlated with precipitation (*r* = −0.61, *P* = 0.04) highlighting the fact that the Baja California flora west of the Gulf occurs in drier environments than the Sonoran flora in the east.

When the distribution patterns of the 476 plant taxa are analyzed, five distinct groups of species can be recognized from their loadings in the first two PCA axes (Fig. 4): small or single island species, generalist species, and large-island species can be distinguished along axis 1, while Sonoran-side species, and Baja-side species clearly separate along axis 2.

There was a high level of nestedness of the small island flora in the large islands, especially with Tiburón where Baselga’s (2010) β_NES_ was 0.68–0.80. Indeed, with the exception of two taxonomically suspect species (an undescribed *Mammillaria* on Dátil and Cholludo islands and *Johnstonella grayi* var. *cryptochaeta* in Salsipuedes, reported in Case & Cody (1983a) but not supported by herbarium specimens), all the rest of the small island flora is nested within the plant list of the large islands. Under the hypothesis of random colonization from the large islands, the Monte–Carlo test predicted a mean Sorensen similarity of 0.04 (s.e. ± 0.04) between small islands, while the true mean similarity between small islands is almost ten times higher (0.34 se ± 0.02). The significant difference (*t* = 6.7, d.f. 3, *P* = 0.003) between the observed similarity among small islands and the much lower value predicted under the random colonization hypothesis endorses the idea that the group of species colonizing small islands is not a random subset of the flora of the larger islands but rather part of a smaller group of species that are able to tolerate the harsh environmental conditions of small islands, like saline sea-spray and eutrophic guano substrates.

The Cactaceae showed a significantly higher richness on the smaller islands compared to the rest of the flora (*X*^2^ = 71.9, d.f. 13, *P* < 0.0001). Most of the small bird-guano islands (Alcatraz, Cholludo, Las Ánimas, Patos, Rasa, Salsipuedes, and San Pedro Mártir) showed a very high proportion of cacti and halophytic succulents (as defined by Wilder, Felger & Romero-Morales (2008)) pooled together, which differed significantly from the overall pattern of species richness (*X*^2^ = 44.8, d.f. 13, *P* < 0.0001). The family Polygonaceae had no species on small and medium islands and only one species (*Eriogonum inflatum*, the common desert trumpet) on large islands (Tiburón, Esteban, and San Lorenzo), but eight species on the otherwise species-poor Ángel de la Guarda, a significantly higher number compared to the other large islands (*X*^2^ = 13.2, d.f. 3, *P* = 0.004).

## Discussion

The theory of island biogeography, especially when analyzed with diverse data sources, continues to serve as a base for understanding patterns of biodiversity. We were able to expand the standard analyses to (1) identify islands that deviate from the model’s predictions and test factors beyond area that have an incidence on species diversity, and (2) explore the composition of the individual island floras to better understand their origin. Despite large-scale transformation of ecosystems in Mexico’s northwest (González-Abraham et al., 2015), diversity levels of the Midriff Islands are still in large part governed by the geologic legacy of the opening of the Gulf of California (Dolby et al., 2015).

### Influences on species richness

Plant species richness patterns among the islands confirms the well-established species-area power function. The ecological heterogeneity of the islands, expressed both as the number of different habitats and topographic variation, accounted for the largest proportion of variation in species richness not accounted by area alone (Table 3). The combination of these two factors in supporting diversity has been long recognized (Darwin, 1860; Preston, 1962; Hortal et al., 2009), including prior analyses for these islands (Felger & Lowe, 1976; Cody, Moran & Thompson, 1983).

The geologic history of the Gulf of California is reflected in the island type: oceanic islands were either formed from the eruption of deep underwater volcanoes or were derived from segments of the peninsular crust that became separated from the peninsula by deep faults millions of years ago. In contrast, landbridge islands are really topographic mounds separated from the coast by shallow channels less than 100 m deep, which were connected to the mainland during most of the Pleistocene glaciations and only became isolated toward the end of the last glacial period, some 10,000 years ago (Graham, Dayton & Erlandson, 2003; Fig. 1). In agreement with the region’s geologic history, landbridge islands have significantly more species than oceanic ones.

An interesting case study is Isla Ángel de la Guarda, an extremely arid oceanic island rifted off the Baja California peninsula ca. two Ma (Nagy & Stock, 2000). Ángel harbors only 217 species, 54 less than predicted by our species-area model and significantly less than the 349 species in the similarly-sized landbridge Tiburón Island that receives almost twice as much rainfall (Felger, Wilder & Romero-Morales, 2012). The effects of isolation on Ángel de la Guarda are also evident in its relatively high level of endemic reptiles, rodents (Lawlor et al., 2002), and plants (Moran, 1983).

Although the geologic nature of the island (landbridge vs. oceanic) and xeromorphic gradient are strongly related to species richness, the distance to the mainland per se did not show a significant relationship with plant species richness. This was true even among oceanic islands (Table 3), likely because of the narrowness of the Gulf. The importance of distance in the Gulf of California is taxon-dependent and varies with colonization and persistence rates (Case & Cody, 1987). Taxa that have high colonization rates, such as birds, show few effects of insularity (Cody & Velarde, 2002), while mammals and reptiles are less dispersive and more often evolve unique forms in isolation (Soulé & Sloan, 1966; Lawlor et al., 2002). Plants, which also have high colonization rates and often possess long distance dispersal ability, show a relatively low level of endemism in these islands, except among Cactaceae (Rebman, 2002).

Finally, the presence of seabird rookeries explains a significant amount of the variation in species numbers: small guano islands harbor less species than similarly-sized islands that lack rookeries. Recent studies have shown that excess nitrogen can significantly reduce the diversity of terrestrial ecosystems (Simkin et al., 2016).

The relatively high value of the species-area exponent (*z*) is the statistical result of the existence of many small guano islands with low species richness. The species composition of these small islands is quite different from that of larger islands or from that of the mainland source (see next section). Porous land-sea boundaries facilitate the movement of nutrients from the ocean into the islands. The importance of marine inputs in regions with high primary productivity on small islands is accentuated by their inherent high perimeter-to-area ratio (Anderson & Wait, 2001; Talley, Huxel & Holyoak, 2006). Accordingly, marine subsidies (in this case guano), have a disproportionate importance as a driver of biological diversity on small islands (McCauley et al., 2012).

### Influences on composition

The small bird islands in our analyses are linked to the productive waters of the Gulf of California through a long chain of ecological interactions. These isolated islands are free of predatory mammals (Lawlor et al., 2002), which combined with shallow relief provide ideal habitat for some of the largest aggregations of breeding seabirds in North America (Velarde et al., 2005). Seabird diets consist mostly of pelagic fish that occur in abundance in the Midriff region and feed on microscopic plankton in extremely productive upwelling zones (Velarde, Ezcurra & Anderson, 2013).

Seabirds deposit copious amounts of marine derived nutrient rich guano, high in nitrogen (N) and phosphorus (P; Hutchinson, 1950), in much greater concentration on bird islands than on non-bird islands in the Gulf of California (Anderson & Polis, 1999). The presence of elevated levels of N and P on seabird islands act as a strong filter that selects for specific plant species, as shown in the high mean Sorensen similarity value of small islands and the identification of a clustering of small/single island species in the PCA and species cladogram analyses (Fig. 4). It is primarily these, and only these, species that occur on the small islands, resulting in the depressed plant diversity observed on these islands. However, the plant species that can tolerate these nutrient loads, such as cacti and halophytic succulents, occur in greater proportion and remarkable abundances (see the insets of Cholludo and San Pedro Mártir islands in Fig. 1). The percentage of succulent and halophilous flora of bird islands in the Midriff region is relatively greater than that on large islands, suggesting a difference in establishment ability within the flora and providing an outstanding example of marine-based nutrients controlling terrestrial diversity patterns in small islands.

Analysis of the insular floras as a matrix reveals the importance of the proportional distance of the larger islands to either the Baja California peninsula and the Sonoran mainland, as well as the E–W precipitation gradient of the Midriff as a driver of species composition (Felger, Wilder & Romero-Morales, 2012). The majority of the species encountered on Ángel have peninsular affinities and are adapted to extreme aridity. Persistent cold-water upwelling and location of Ángel on the western edge of the North American monsoon, as well as the southeastern edge of Pacific-derived winter storms result in the western Midriff Islands being the most arid portions of the Gulf of California. In addition, ca. 30 plant taxa characteristic of the northern mediterranean region of the Peninsula reach their southern limit on or near Ángel (Moran, 1983). This is exemplified in our analysis by the preponderance of the buckwheat family Polygonaceae, a family that is strongly associated to the California Floristic Province, on Ángel and its near absence on Tiburón.

### The human dimension

Humans have been a broad-scale determinant of species diversity on islands worldwide, often through habitat disruptions and species extinctions (Burney & Flannery, 2005) and cultural use (Bye & Linares, 2000; Heinsohn, 2003). Portions of the world thought to be pristine, without human modification, have been shown to be otherwise (Denevan, 1992; Gómez-Pompa & Kaus, 1992). While our analysis is limited to the presence/absence of archaeological elements on the islands, careful ethnographic work with the Comcaac (Seri people) on the coast of Sonora has helped illuminate their profound interaction with the island and coastal environments (Felger, 1976; Nabhan, 2003; Wilder et al., 2016). The consumption, utilitarian use, harvesting, and transport of plants across the region and between islands by the Comcaac and or their ancestors has been shown repeatedly (Felger & Moser, 1985; Nabhan, 2002).

The unique culture of the Comcaac grew and evolved in the Gulf of California, and is endemic to the region. Their language, *Cmiique Iitom* is a language isolate (Moser & Marlett, 2010) and their knowledge of the natural world is extensive (Felger & Moser, 1985; Marlett, 2014). They lived in and visited the larger Gulf of California region for thousands of years, with archaeological evidence of their presence on many islands in the Midriff (Table 1; Bowen, 2000, 2009). Yet, any environmental changes wrought by the Comcaac have been integrated with and are indistinguishable from the background ecological dynamics of the Midriff Islands. This is most likely because human population pressure was low and varied from ca. 180–3,500 individuals through time (Nabhan, 2002), limited by the scarcity of fresh water (Felger & Moser, 1985).

One manifestation of the non-altered environment of the Midriff Islands is the low frequency of non-native plant species (Felger & Moser, 1985; Felger, Wilder & Romero-Morales, 2012). Just 2.5% (12 species) of the Midriff Island flora is non-native (Wilder, 2014), relative to non-native floristic compositions of 14% among the Baja California Pacific Islands and 28% among the California Channel Islands (Ratay, Vanderplank & Wilder, 2014). The diminished presence of foreign cultures in the homeland of the Comcaac greatly reduced the opportunities for the establishment of non-native species.

## Conclusions

Our results support what is possibly the most important tenet of the equilibrium theory of island biogeography, namely that the power function for area predicts most of the variation observed in species richness. We also found the power exponent *z* to be significantly larger than 0.263, Preston’s canonical value, a fact that is commonly reported in island studies. More interestingly, the analysis of the residual variation in species richness, unexplained by the islands’ area, yielded important additional insights. The ecological heterogeneity of the islands, expressed both as the number of different habitats and topographic variation, accounts for much of the total variation in species richness. Marine derived guano substrates on oceanic islands harbor fewer species than guano-free substrates or landbridge islands. It is likely the increase in aridity in the Gulf from east to west also plays a role in limiting diversity.

The species composition of the islands varies significantly with island area. Small islands have a particular flora of their own, highly adapted to maritime environments and guano deposition, and cannot be considered a random subset of the continental floras. The flora of the larger islands, on the other hand, is strongly dependent on the continental source and the prevailing precipitation patterns. Islands near the Sonoran coast showed a largely Sonoran flora, while Islands near the Baja California peninsula were much drier and showed a Baja Californian floristic assemblage.

Islands with ecological networks that function as they were operating hundreds of years ago are rare, and islands where human presence has not irreversibly modified the native biological diversity are scarcer yet. During the 20th century, large-scale settlements and extractive resource have irreversibly modified continental and peninsular landscapes around the Gulf of California. In that perspective, the insular biota of the Midriff Islands represents a set of relictual ecosystems, the best conserved portion of the Sonoran Desert. Step-wise testing with modern regression methods of the potential primary drivers of species diversity and composition in these islands allowed us to identify the main factors controlling species richness and to establish a botanical baseline for these important ecosystems. This knowledge, in turn, is critical in the face of widespread extinctions and ecological chains increasingly shifting to a biodiversity-impoverished state with reduced baselines (Dayton et al., 1998; Sáenz-Arroyo et al., 2005; McCauley et al., 2012). Taken as a whole, our data highlights the unique conservation status of a set of desert microcosms whose biodiversity is still largely unaffected by human action where the main tenets of insular ecology can be put to a test.

## Supplemental Information

10.7717/peerj.7286/supp-1 Supplemental Information 1 Checklist for the flora of the Midriff Islands.

10.7717/peerj.7286/supp-2 Supplemental Information 2 Alternative derivation of the species-area curve.

## Additional Information and Declarations

### Field Study Permissions

The following information was supplied relating to field study approvals (i.e., approving body and any reference numbers): Collections were made under Mexican federal collecting permit issued by SEMARNAT to Dr. Ezcurra (NOM-126-SEMARNAT-2000).

### Data Availability

The following information was supplied regarding data availability: Floristic data associated with this manuscript are available in Appendix S1 and available via search by region or plant species in SEINet: http://swbiodiversity.org/seinet/.

## References

[ref-1] Anderson WB, Polis GA (1999). Nutrient fluxes from water to land: seabirds affect plant nutrient status on Gulf of California islands. Oecologia.

[ref-2] Anderson WB, Wait DA (2001). Subsidized island biogeography hypothesis: another new twist on an old theory. Ecology Letters.

[ref-3] Angiosperm Phylogeny Group (2016). An update of the Angiosperm Phylogeny Group classification for the orders and families of flowering plants: APG IV. Botanical Journal of the Linnean Society.

[ref-4] Aragón-Arreola M, Martín-Barajas A (2007). Westward migration of extension in the northern Gulf of California, Mexico. Geology.

[ref-5] Barrett K, Wait DA, Anderson WB (2003). Small island biogeography in the Gulf of California: lizards, the subsidized island biogeography hypothesis, and the small island effect. Journal of Biogeography.

[ref-6] Baselga A (2010). Partitioning the turnover and nestedness components of beta diversity. Global Ecology and Biogeography.

[ref-7] Bowen T (2000). Unknown Island: Seri Indians, Europeans, and San Esteban Island in the Gulf of California.

[ref-8] Bowen T (2009). The record of native people on Gulf of California islands.

[ref-9] Brown JH, West GB (2000). Scaling in biology.

[ref-10] Burney DA, Flannery TF (2005). Fifty millennia of catastrophic extinctions after human contact. Trends in Ecology and Evolution.

[ref-11] Búrquez A, Martínez-Yrízar A, Felger RS, Yetmanm D, Robichaux RH (1999). Vegetation and habitat diversity at the southern edge of the Sonoran Desert. Ecology of Sonoran Desert Plants and Plant Communities.

[ref-12] Bye R, Linares E, Minnis PE, Elisens WJ (2000). Relationships between Mexican ethnobotanical diversity and indigenous peoples. Biodiversity and Native America.

[ref-13] Carreño AL, Helenes J, Case J, Cody ML, Ezcurra E (2002). Geology and ages of the islands. A New Island Biogeography of the Sea of Cortés.

[ref-14] Case TJ, Cody ML (1983a). Island biogeography in the Sea of Cortéz.

[ref-15] Case TJ, Cody ML, Case TJ, Cody ML (1983b). Synthesis: pattern and processes in island biogeography. Island Biogeography in the Sea of Cortéz.

[ref-16] Case TJ, Cody ML (1987). Testing theories of island biogeography. American Scientist.

[ref-17] Case TJ, Cody ML, Ezcurra E (2002). A new island biogeography of the sea of Cortés.

[ref-18] Cody ML, Moran R, Thompson H, Case TJ, Cody ML (1983). The plants. Island Biogeography in the Sea of Cortéz.

[ref-19] Cody ML, Velarde E, Case J, Cody ML, Ezcurra E (2002). Land birds. A New Island Biogeography of the Sea of Cortés.

[ref-20] CONAGUA (2010). Determinación de la disponibilidad de agua en el acuífero 2617 Puerto Libertad, Estado de Sonora.

[ref-21] Darwin CR (1860). Journal of researches into the natural history and geology of the countries visited during the voyage of H.M.S. Beagle round the world, under the command of Capt. Fitz Roy R.N.

[ref-22] Davis LG, Laylander D, Moore JD (2006). Baja California’s paleoenvironmental context. The Prehistory of Baja California.

[ref-23] Dayton PK, Tegner MJ, Edwards PB, Riser KL (1998). Sliding baselines, ghosts, and reduced expectations in kelp forest communities. Ecological Applications.

[ref-24] Denevan WM (1992). The pristine myth: the landscape of the Americas in 1492. Annals of the Association of American Geographers.

[ref-25] Desonie DL (1992). Geologic and geochemical reconnaissance of Isla San Esteban: post-subduction orogenic volcanism in the Gulf of California. Journal of Volcanology and Geothermal Research.

[ref-26] DOF, Diario Oficial de la Federación (2000). Decreto del 7 de junio. Declara el Área de Protección de Flora y Fauna “Islas del Golfo de California”.

[ref-27] Dolby G, Bennett SEK, Lira-Noriega A, Wilder BT, Munguia-Vega A (2015). The geologic and climatic forcing of biodiversity surrounding the Gulf of California. Journal of the Southwest.

[ref-28] Douglas R, Gonzalez-Yajimovich O, Ledesma-Vazquez L, Staines-Urias F (2007). Climate forcing, primary production and the distribution of Holocene biogenic sediments in the Gulf of California. Quaternary Science Reviews.

[ref-29] Duberstein JN, Jimenez-Serrania V, Pfister TA, Lindquist KE, Meltzer L, Ralph CJ, Rich TD (2005). Breeding double-crested cormorants and wading birds on Isla Alcatraz, Sonora, México. Bird Conservation Implementation and Integration in the Americas: Proceedings of the Third International Partners in Flight Conference.

[ref-30] Duffy DE (1990). On continuity-corrected residuals in logistic regression. Biometrika.

[ref-31] Felger RS (1966). Ecology of the Islands and Gulf Coast of Sonora, Mexico.

[ref-32] Felger RS (1976). Gulf of California—an ethno-ecological perspective. Natural Resources Journal.

[ref-33] Felger RS, Lowe CH (1976). The island and coastal vegetation and flora of the northern part of Gulf of California. Natural History Museum of Los Angeles County, Contributions in Science.

[ref-34] Felger RS, Moser MB (1985). People of the desert and Sea: ethnobotany of the Seri Indians.

[ref-35] Felger RS, Wilder BT, Gallo-Reynoso JP (2011). Floristic diversity and long-term vegetation dynamics of Isla San Pedro Nolasco, Gulf of California, Mexico. Proceedings of the San Diego Society of Natural History.

[ref-36] Felger RS, Wilder BT, Romero-Morales H (2012). Plant life of a desert archipelago: flora of the Sonoran Islands in the Gulf of California.

[ref-37] García E, Vidal R, Tamayo LM, Reyna T, Sánchez R, Soto M, Soto E (1973). Precipitacion y probabilidad de lluvia en el estado de Sonora. Comisión de Estudios del Territorio Nacional (CETENAL).

[ref-38] Gentry HS (1949). Land plants collected by the Vallero III, Allan Hancock Pacific Expeditions 1937–1951.

[ref-39] Gómez-Pompa A, Kaus A (1992). Taming the wilderness myth. BioScience.

[ref-40] González-Abraham C, Ezcurra E, Garcillán PP, Ortega-Rubio A, Kolb M, Bezaury-Creel JE (2015). The human footprint in Mexico: physical geography and historical legacies. PLOS ONE.

[ref-41] Gould SJ (1979). An allometric interpretation of species-area curves: the meaning of the coefficient. American Naturalist.

[ref-42] Graham MH, Dayton PK, Erlandson JM (2003). Ice ages and ecological transitions on temperate coasts. Trends in Ecology and Evolution.

[ref-43] Heinsohn T (2003). Animal translocation: long-term human influences on the vertebrate zoogeography of Australasia (natural dispersal versus ethnophoresy). Australian Zoologist.

[ref-44] Hortal J, Triantis KA, Meiri S, Thébault E, Sfenthourakis S (2009). Island species richness increases with habitat diversity. American Naturalist.

[ref-45] Hutchinson GE (1950). The biogeochemistry of vertebrate excretion. Bulletin of the American Museum of Natural History.

[ref-46] Jackson DA (1993). Stopping rules in principal components analysis: a comparison of heuristical and statistical approaches. Ecology.

[ref-47] Johnston IM (1924). Expedition of the California Academy of Sciences to the Gulf of California in 1921: the botany (vascular plants). Proceedings of the California Academy of Sciences IV.

[ref-48] Lambeck K, Chappell J (2001). Sea level change through the last glacial cycle. Science.

[ref-49] Lawlor TE, Hafner DJ, Stapp P, Riddle BR, Alvarez-Castañeda ST, Case TJ, Cody ML, Ezcurra E (2002). The mammals. A New Island Biogeography of the Sea of Cortés.

[ref-50] Ledesma-Vázquez J, Carreño AL, Brusca R (2010). Origin, Age, and geological evolution of the Gulf of California. The Gulf of California: Biodiversity and Conservation.

[ref-51] Lindsay GE (1955). Notes concerning the botanical explorers and expedition of Lower California, Mexico.

[ref-52] Lindsay GE (1966). The Gulf Islands expedition of 1966. Proceedings of the California Academy of Sciences IV.

[ref-53] MacArthur RH, Wilson EO (1967). Theory of Island Biogeography.

[ref-54] Marlett CM (2014). Shells on a desert shore: mollusks in the Seri world.

[ref-55] Maya JA (1968). The natural history of the fish-eating bat, *Pizonyx vivesi*.

[ref-56] McCauley DJ, Desalles PA, Young HS, Dunbar RB, Dirzo R, Mills MM, Micheli F (2012). From wing to wing: the persistence of long ecological interaction chains in less-disturbed ecosystems. Scientific Reports.

[ref-57] McCullagh P, Nelder JA (1989). Generalized linear models.

[ref-58] Moran R, Case TJ, Cody ML (1983). The vascular flora of Isla Ángel de la Guarda. Island Biogeography of the Sea of Cortéz.

[ref-59] Moser MB, Marlett SA (2010). Comcaac quih yaza quih hant ihiip hac; Diccionario seri-español-inglés.

[ref-60] Murphy RW, Sanchez-Piñero F, Polis GA, Aalbu RL, Case TJ, Cody ML, Ezcurra E (2002). New measurements of area and distance for islands in the Sea of Cortés. A New Island Biogeography of the Sea of Cortés.

[ref-61] Nabhan GP, Case TJ, Cody ML, Ezcurra E (2002). Cultural dispersal of plants and reptiles. A New Island Biogeography of the Sea of Cortés.

[ref-62] Nabhan GP (2003). Singing the turtles to sea: the Comcáac (Seri) art and science of reptiles.

[ref-63] Nagy EA, Stock JM (2000). Structural controls on the continent-ocean transition in the northern Gulf of California. Journal of Geophysical Research: Solid Earth.

[ref-64] Noy-Meir I (1973). Divisive polythetic classification of vegetation data by optimized division on ordination components. Journal of Ecology.

[ref-65] Patiño J, Whittaker RJ, Borges PAV, Fernández-Palacios JM, Ah-Peng C, Araújo MB, Ávila SP, Cardoso P, Cornuault J, De Boer EJ, De Nascimento L, Gil A, González-Castro A, Gruner DS, Heleno R, Hortal J, Illera JC, Kaiser-Bunbury CN, Matthews TJ, Papadopoulou A, Pettorelli N, Price JP, Santos AMC, Steinbauer MJ, Triantis KA, Valente L, Vargas P, Weigelt P, Emerson BC (2017). A roadmap for island biology: 50 fundamental questions after 50 years of The Theory of Island Biogeography. Journal of Biogeography.

[ref-66] Polis GA, Hurd SD, Jackson CT, Sanchez-Piñero F (1997). El Niño effects on the dynamics and control of an island ecosystem in the Gulf of California. Ecology.

[ref-67] Preston FW (1962). The canonical distribution of commonness and rarity: Part I. Ecology.

[ref-68] R Core Team (2016). R: a language and environment for statistical computing.

[ref-69] Ratay SE, Vanderplank SE, Wilder BT (2014). Island specialists: shared flora of the Alta and Baja California Pacific Islands. Monographs of the Western North American Naturalist.

[ref-70] Rebman JP, Case TJ, Cody ML, Ezcurra E (2002). Plants endemic to the Gulf Islands. A New Island Biogeography of the Sea of Cortés.

[ref-71] Rebman JP (2010). A vascular plant checklist of Isla Ángel de la Guarda, Baja California, Mexico. San Diego Natural History Museum. http://bajaflora.org/Floras/AngeldelaGuarda.html.

[ref-72] Rebman JP, León de la Luz JL, Moran RV, Case TJ, Cody ML, Ezcurra E (2002). Vascular plants of the Gulf Islands. A New Island Biogeography of the Sea of Cortés.

[ref-73] Ruiz Corral JA, Diaz Padilla G, Guzman Ruiz SD, Medina Garcia G, Silva Serna MM (2006a). Estadísticas climatológicas básicas del estado de Baja California (Período 1961–2003).

[ref-74] Ruiz Corral JA, Medina Garcia G, Grageda Grageda J, Silva Serna MM, Diaz Padilla G (2005). Estadisticas climatologicas basicas del estado de Sonora (Período 1961–2003).

[ref-75] Ruiz Corral JA, Medina Garcia G, Meza Sanchez R, Diaz Padilla G, Serrano al Tamirano V (2006b). Estadísticas climatológicas básicas del estado de Baja California Sur (Período 1961–2003).

[ref-76] Sáenz-Arroyo A, Roberts CM, Torre J, Cariño-Olvera M, Enríquez-Andrade RR (2005). Rapidly shifting environmental baselines among fishers of the Gulf of California. Proceedings of the Royal Society B: Biological Sciences.

[ref-77] Sanchez-Piñero F, Polis GA (2000). Bottom-Up dynamics of allochthonous input: direct and indirect effects of seabirds on Islands. Ecology.

[ref-78] Santos AM, Field R, Ricklefs RE (2016). New directions in island biogeography. Global Ecology and Biogeography.

[ref-79] Sfenthourakis S, Triantis KA (2009). Habitat diversity, ecological requirements of species and the Small Island Effect. Diversity and Distributions.

[ref-80] Shreve F (1951). Vegetation of the Sonoran Desert.

[ref-81] Simkin SM, Allen EB, Bowman WD, Clark CM, Belnap J, Brooks ML, Cade BS, Collins SL, Geiser LH, Gilliam FS, Jovan SE, Pardo LH, Schulz BK, Stevens CJ, Suding KN, Throop HL, Waller DM (2016). Conditional vulnerability of plant diversity to atmospheric nitrogen deposition across the United States. Proceedings of the National Academy of Sciences of the United States of America.

[ref-82] Soberón J, Llorente J (1993). The use of species accumulation functions for the prediction of species richness. Conservation Biology.

[ref-83] Soulé M, Sloan AJ (1966). Biogeography and distribution of the reptiles and amphibians on islands in the Gulf of California, Mexico. Transactions of the San Diego Society of Natural History.

[ref-84] Stevens PF (2019). www.mobot.org/MOBOT/research/APweb/.

[ref-85] Stock JM (2000). Relation of the Puertecitos Volcanic Province, Baja California, Mexico, to development of the plate boundary in the Gulf of California. Geological Society of America Special Papers.

[ref-86] Talley DM, Huxel GR, Holyoak M, Crooks KR, Sanjayan M (2006). Connectivity at the land-water interface. Connectivity Conservation.

[ref-87] Tershy BR, Breese D (1997). The birds of San Pedro Mártir Island, Gulf of California, Mexico. Western Birds.

[ref-88] Townsend CH (1916). Voyage of the “Albatross” to the Gulf of California in 1911. Bulletin of the American Museum of Natural History.

[ref-89] Velarde E (1989). Conducta y ecología de la reproducción de la gaviota parda (*Larus heermanni*) en Isla Rasa, Baja California. Tesis de Doctorado en Ciencias (Biología), Mexico, D.F: Facultad de Ciencias, Universidad Nacional Autónoma de México.

[ref-90] Velarde E, Ceballos G, Valdemar LM (2000). Paiño mínimo (*Oceanodroma microsoma*). Las aves de México en peligro de extinction.

[ref-91] Velarde E, Cartron JLE, Drummond H, Anderson DW, Gallardo FR, Palacios E, Rodríguez C, Cartron JLE, Ceballos G, Felger RS (2005). Nesting seabirds of the Gulf of California’s Offshore islands: diversity, ecology and conservation. Biodiversity, Ecosystems, and Conservation in Northern Mexico.

[ref-92] Velarde E, Ezcurra E, Anderson DW (2013). Seabird diets provide early warning of sardine fishery declines in the Gulf of California. Scientific Reports.

[ref-93] Velarde E, Wilder BT, Felger RS, Ezcurra E (2014). Floristic diversity and dynamics of Isla Rasa, Gulf of California—a globally important seabird island. Botanical Sciences.

[ref-94] Wallace AR (1880). Island life.

[ref-95] Warren BH, Simberloff D, Ricklefs RE, Aguilée R, Condamine FL, Gravel D, Morlon H, Mouquet N, Rosindell J, Casquet J, Conti E, Cornuault J, Fernández-Palacios JM, Hengl T, Norder SJ, Rijsdijk KF, Sanmartín I, Strasberg D, Triantis KA, Valente LM, Whittaker RJ, Gillespie RG, Emerson BC, Thébaud C (2015). Islands as model systems in ecology and evolution: prospects fifty years after MacArthur-Wilson. Ecology Letters.

[ref-96] Whitehead DR, Jones CE (1969). Small islands and the equilibrium theory of insular biogeography. Evolution.

[ref-97] Wilcox BA (1978). Supersaturated island faunas: a species-age relationship for lizards on post-Pleistocene land-bridge islands. Science.

[ref-98] Wilder BT (2014). Historical biogeography of the Midriff Islands in the Gulf of California, Mexico.

[ref-99] Wilder BT, Felger RS (2010). Dwarf giants, guano, and isolation: vegetation and floristic diversity of Isla San Pedro Mártir, Gulf of California, Mexico. Proceedings, San Diego Natural History Museum.

[ref-100] Wilder BT, Felger RS, Romero-Morales H (2008). Succulent plant diversity of the Sonoran Islands, Gulf of California, Mexico. Haseltonia.

[ref-101] Wilder BT, O’Meara C, Monti L, Nabhan GP (2016). The importance of indigenous knowledge in curbing the loss of language and biodiversity. BioScience.

