# Supplementary material for: Controls of plant diversity and composition on a desert archipelago
Source: PeerJ. 2019 Jul 9;7:e7286. doi: 10.7717/peerj.7286 (PMC6625499; doi:10.7717/peerj.7286)
Supplement: Supplemental Information 1 [file peerj-07-7286-s001.docx]

SUPPORTING INFORMATION

Controls of plant diversity and composition on a desert archipelago

Benjamin T. Wilder, Richard S. Felger, Exequiel Ezcurra

**Supplemental Appendix S1. Checklist for the flora of the Midriff Islands**

* = non-native species; (?) = occurrence uncertain, though accepted and included in analysis; (obs) = presence based on observation only and included in analysis.

| **FAMILY** | **SPECIES** | **Tiburón** | **Ángel** | **Esteban** | **San Lorenzo** | **Las Ánimas** | **Nolasco** | **Mártir** | **Alcatraz** | **Partidad Norte** | **Dátil** | **Salsipuedes** | **Rasa** | **Patos** | **Cholludo** |
| --- | --- | --- | --- | --- | --- | --- | --- | --- | --- | --- | --- | --- | --- | --- | --- |
| Acanthaceae | *Avicennia germinans* | TIB |  |  |  |  |  |  | ALC |  |  |  |  |  |  |
| Acanthaceae | *Carlowrightia arizonica* | TIB | ANG | EST |  |  |  |  |  |  | DAT |  |  |  |  |
| Acanthaceae | *Dicliptera resupinata* | TIB |  |  |  |  |  |  |  |  |  |  |  |  |  |
| Acanthaceae | *Elytraria imbricata* | TIB |  |  |  |  |  |  |  |  |  |  |  |  |  |
| Acanthaceae | *Holographis virgata* subsp. *virgata* | TIB |  |  |  |  |  |  |  |  | DAT |  |  |  |  |
| Acanthaceae | *Justicia californica* | TIB | ANG | EST |  |  |  |  | ALC |  |  |  |  |  | CHO |
| Acanthaceae | *Justicia candicans* | TIB |  |  |  |  |  |  |  |  |  |  |  |  |  |
| Acanthaceae | *Justicia longii* | TIB |  |  |  |  |  |  |  |  |  |  |  |  |  |
| Acanthaceae | *Ruellia californica* | TIB |  |  |  |  |  |  |  |  |  |  |  |  |  |
| Acanthaceae | *Tetramerium fruticosum* | TIB |  |  |  |  |  |  |  |  |  |  |  |  |  |
| Achatocarpaceae | *Phaulothamnus spinescens* | TIB |  |  |  |  | NOL |  | ALC |  | DAT |  |  |  | CHO |
| Aizoaceae | *Mesembryanthemum crystallinum** |  |  |  |  |  |  |  |  |  | DAT |  |  |  |  |
| Aizoaceae | *Sesuvium portulacastrum* | TIB | ANG |  | LOR |  |  |  | ALC |  |  |  | RAS |  |  |
| Aizoaceae | *Trianthema portulacastrum* | TIB | ANG |  |  | ANI |  | MAR | ALC |  |  |  |  | PAT |  |
| Alliaceae | *Allium haematochiton* |  | ANG |  |  |  |  |  |  |  |  |  |  |  |  |
| Amaranthaceae | *Allenrolfea occidentalis* | TIB | ANG |  |  |  |  |  | ALC |  |  |  |  |  |  |
| Amaranthaceae | *Amaranthus fimbriatus* | TIB | ANG |  |  |  | NOL | MAR | ALC |  | DAT |  |  | PAT |  |
| Amaranthaceae | *Amaranthus watsonii* | TIB | ANG | EST | LOR | ANI |  |  | ALC | PAR | DAT | SAL |  |  |  |
| Amaranthaceae | *Arthrocnemum subterminale* | TIB | ANG |  |  |  |  |  | ALC |  |  |  | RAS |  |  |
| Amaranthaceae | *Atriplex barclayana* | TIB | ANG | EST | LOR | ANI |  |  | ALC | PAR | DAT | SAL | RAS | PAT | CHO |
| Amaranthaceae | *Atriplex hymenelytra* |  | ANG |  |  |  |  |  |  |  |  |  |  |  |  |
| Amaranthaceae | *Atriplex linearis* | TIB |  |  | LOR |  |  |  |  |  |  |  |  |  |  |
| Amaranthaceae | *Atriplex polycarpa* | TIB | ANG | EST | LOR |  |  |  |  |  | DAT |  |  |  |  |
| Amaranthaceae | *Chenopodiastrum murale** | TIB | ANG |  |  |  |  |  | ALC |  |  |  |  | PAT | CHO |
| Amaranthaceae | *Salicornia bigelovii* | TIB | ANG |  |  |  |  |  |  |  |  |  |  |  |  |
| Amaranthaceae | *Suaeda esteroa* | TIB |  |  |  |  |  |  |  |  |  |  |  |  |  |
| Amaranthaceae | *Suaeda nigra* | TIB |  | EST |  |  |  |  | ALC |  |  |  |  | PAT |  |
| Amaranthaceae | *Suaeda taxifolia* |  | ANG |  |  |  |  |  |  |  |  |  |  |  |  |
| Amaranthaceae | *Tidestromia lanuginosa* subsp. e*liassoniana* | TIB |  |  |  |  |  |  |  |  |  |  |  |  |  |
| Anacardiaceae | *Pachycormus discolor* |  | ANG |  |  |  |  |  |  |  |  |  |  |  |  |
| Anacardiaceae | *Rhus kearneyi* subsp. *borjaensis* |  | ANG |  |  |  |  |  |  |  |  |  |  |  |  |
| Apiaceae | *Apiastrum angustifolium* |  | ANG |  |  |  |  |  |  |  |  |  |  |  |  |
| Apiaceae | *Daucus pusillus* | TIB |  |  |  |  |  |  |  |  |  |  |  |  |  |
| Apocynaceae | *Asclepias albicans* | TIB | ANG | EST | LOR |  |  |  |  |  | DAT |  |  |  |  |
| Apocynaceae | *Asclepias subulata* | TIB |  |  |  |  |  |  |  |  |  |  |  |  |  |
| Apocynaceae | *Funastrum heterophyllum* | TIB |  |  |  |  |  |  |  |  |  |  |  |  |  |
| Apocynaceae | *Haplophyton cimicidum* | TIB |  |  |  |  |  |  |  |  |  |  |  |  |  |
| Apocynaceae | *Marsdenia edulis* | TIB |  |  |  |  |  |  |  |  |  |  |  |  |  |
| Apocynaceae | *Matelea hastulata* |  | ANG |  |  |  |  |  |  |  |  |  |  |  |  |
| Apocynaceae | *Matelea pringlei* | TIB |  |  |  |  |  |  |  |  | DAT |  |  |  |  |
| Apocynaceae | *Metastelma arizonicum* | TIB |  |  |  |  |  |  |  |  |  |  |  |  |  |
| Apocynaceae | *Metastelma californicum* |  |  |  |  |  | NOL |  |  |  |  |  |  |  |  |
| Apocynaceae | *Polystemma cordifolium* | TIB |  |  |  |  |  |  |  |  |  |  |  |  |  |
| Apocynaceae | *Vallesia glabra* | TIB |  |  |  |  |  |  |  |  |  |  |  |  |  |
| Arecaceae | *Brahea armata* | TIB | ANG |  |  |  |  |  |  |  |  |  |  |  |  |
| Aristolochiaceae | *Aristolochia watsonii* | TIB |  |  |  |  |  |  |  |  |  |  |  |  |  |
| Asparagaceae | *Agave cerulata* subsp. *cerulata* |  | ANG |  |  |  |  |  |  |  |  |  |  |  |  |
| Asparagaceae | *Agave cerulata* subsp. *dentiens* |  |  | EST |  |  |  |  |  |  |  |  |  |  |  |
| Asparagaceae | *Agave chrysoglossa* | TIB |  |  |  |  | NOL |  |  |  |  |  |  |  |  |
| Asparagaceae | *Agave* sp. | TIB |  |  |  |  |  |  |  |  |  |  |  |  |  |
| Asparagaceae | *Agave subsimplex* | TIB |  |  |  |  |  |  |  |  | DAT |  |  |  | CHO |
| Asparagaceae | *Dasylirion* *gentryi* | TIB |  |  |  |  |  |  |  |  |  |  |  |  |  |
| Asparagaceae | *Nolina bigelovii* |  | ANG |  |  |  |  |  |  |  |  |  |  |  |  |
| Asparagaceae | *Triteleiopsis palmeri* | TIB |  |  |  |  |  |  |  |  |  |  |  |  |  |
| Asteraceae | *Acourtia palmeri* |  | ANG |  |  |  |  |  |  |  |  |  |  |  |  |
| Asteraceae | *Adenophyllum porophylloides* |  | ANG |  |  |  |  |  |  |  |  |  |  |  |  |
| Asteraceae | *Ambrosia × platyspina* |  | ANG |  |  |  |  |  |  |  |  |  |  |  |  |
| Asteraceae | *Ambrosia camphorata* var. *leptophylla* | TIB |  |  |  |  |  |  |  |  |  |  |  |  |  |
| Asteraceae | *Ambrosia carduacea* | TIB |  |  |  |  |  |  |  |  |  |  |  |  |  |
| Asteraceae | *Ambrosia chenopodiifolia* |  | ANG |  |  |  |  |  |  |  |  |  |  |  |  |
| Asteraceae | *Ambrosia divaricata* | TIB |  | EST |  |  |  |  |  |  | DAT |  |  |  |  |
| Asteraceae | *Ambrosia dumosa* | TIB | ANG |  | LOR |  |  |  |  |  |  |  |  |  |  |
| Asteraceae | *Ambrosia ilicifolia* | TIB | ANG | EST | LOR | ANI |  |  |  |  |  |  |  |  |  |
| Asteraceae | *Ambrosia magdalenae* |  | ANG |  |  |  |  |  |  |  |  |  |  |  |  |
| Asteraceae | *Ambrosia salsola* var. *pentalepis* | TIB | ANG |  |  |  |  |  |  |  |  |  |  |  |  |
| Asteraceae | *Baccharis salicifolia* | TIB |  |  |  |  |  |  |  |  |  |  |  |  |  |
| Asteraceae | *Baccharis sarothroides* |  | ANG (obs) |  | LOR |  |  | MAR |  |  |  |  |  |  |  |
| Asteraceae | *Bahiopsis chenopodina* | TIB |  |  |  |  |  |  |  |  | DAT |  |  |  |  |
| Asteraceae | *Bahiopsis triangularis* |  | ANG | EST | LOR |  | NOL |  |  |  |  |  |  |  |  |
| Asteraceae | *Bajacalia crassifolia* | TIB | ANG | EST | LOR |  |  |  |  |  | DAT |  |  |  |  |
| Asteraceae | *Bebbia juncea* var. *aspera* | TIB | ANG | EST | LOR |  | NOL |  |  |  | DAT |  |  |  |  |
| Asteraceae | *Brickellia coulteri* var. *coulteri* | TIB |  |  |  |  |  |  |  |  |  |  |  |  |  |
| Asteraceae | *Chromolaena sagittata* | TIB |  |  |  |  |  |  |  |  |  |  |  |  |  |
| Asteraceae | *Coreocarpus parthenioides* var. *parthenioides* |  | ANG |  |  |  |  |  |  |  |  |  |  |  |  |
| Asteraceae | *Coreocarpus sanpedroensis* |  |  |  |  |  | NOL |  |  |  |  |  |  |  |  |
| Asteraceae | *Coreocarpus sonoranus* | TIB |  |  |  |  |  |  |  |  | DAT |  |  |  |  |
| Asteraceae | *Encelia farinosa* var. *farinosa* | TIB | ANG |  | LOR | ANI |  |  | ALC |  | DAT |  |  | PAT |  |
| Asteraceae | *Encelia farinosa* var. *phenicodonta* |  | ANG |  |  |  |  |  |  |  |  |  |  |  |  |
| Asteraceae | *Gutierrezia microcephala* |  | ANG |  |  |  |  |  |  |  |  |  |  |  |  |
| Asteraceae | *Gutierrezia ramulosa* |  | ANG |  |  |  |  |  |  |  |  |  |  |  |  |
| Asteraceae | *Gymnosperma glutinosum* | TIB |  |  |  |  |  |  |  |  |  |  |  |  |  |
| Asteraceae | *Helianthus niveus* var. *niveus* | TIB |  |  |  |  |  |  |  |  |  |  |  |  |  |
| Asteraceae | *Heliopsis anomala* | TIB |  |  |  |  |  |  |  |  |  |  |  |  |  |
| Asteraceae | *Hofmeisteria crassifolia* |  |  |  |  |  | NOL |  |  |  |  |  |  |  |  |
| Asteraceae | *Hofmeisteria fasciculata* var. *fasciculata* | TIB | ANG | EST | LOR | ANI |  |  |  | PAR | DAT |  |  |  | CHO |
| Asteraceae | *Hofmeisteria filifolia* |  | ANG |  |  |  |  |  |  |  |  |  |  |  |  |
| Asteraceae | *Logfia filaginoides* |  | ANG |  |  |  |  |  |  |  |  |  |  |  |  |
| Asteraceae | *Malperia tenuis* |  | ANG |  |  |  |  |  |  |  |  |  |  |  |  |
| Asteraceae | *Palafoxia arida* var. *arida* | TIB |  |  |  |  |  |  | ALC |  |  |  |  |  |  |
| Asteraceae | *Pectis papposa* var. *papposa* | TIB |  |  |  |  |  |  | ALC |  |  |  |  |  |  |
| Asteraceae | *Pectis rusbyi* | TIB |  |  |  |  |  |  |  |  |  |  |  |  | CHO |
| Asteraceae | *Pelucha trifida* | TIB | ANG |  |  |  |  | MAR |  |  |  |  |  |  |  |
| Asteraceae | *Perityle aurea* | TIB | ANG | EST |  |  |  |  |  |  |  |  |  |  |  |
| Asteraceae | *Perityle californica* |  |  |  | LOR |  | NOL |  |  |  |  |  |  |  |  |
| Asteraceae | *Perityle emoryi* | TIB | ANG | EST | LOR | ANI |  | MAR | ALC | PAR | DAT | SAL |  | PAT | CHO |
| Asteraceae | *Peucephyllum schottii* |  | ANG | EST | LOR |  |  |  |  |  |  |  |  |  |  |
| Asteraceae | *Pleurocoronis laphamioides* | TIB | ANG | EST |  |  | NOL | MAR | ALC |  | DAT | SAL |  |  |  |
| Asteraceae | *Pleurocoronis pluriseta* |  | ANG |  |  |  |  |  |  |  |  |  |  |  |  |
| Asteraceae | *Pluchea salicifolia* | TIB |  |  |  |  |  |  |  |  |  |  |  |  |  |
| Asteraceae | *Porophyllum gracile* | TIB | ANG | EST | LOR |  |  |  |  |  | DAT |  |  |  |  |
| Asteraceae | *Porophyllum pausodynum* |  |  |  |  |  | NOL |  |  |  |  |  |  |  |  |
| Asteraceae | *Senecio mohavensis* |  | ANG |  |  |  |  |  |  |  |  |  |  |  |  |
| Asteraceae | *Stephanomeria pauciflora* | TIB |  |  |  |  |  |  |  |  |  |  |  |  |  |
| Asteraceae | *Thymophylla concinna* | TIB |  |  |  |  |  |  |  |  |  |  |  |  |  |
| Asteraceae | *Trichoptilium incisum* |  | ANG |  |  |  |  |  |  |  |  |  |  |  |  |
| Asteraceae | *Trixis californica* var. *californica* | TIB | ANG | EST | LOR |  | NOL | MAR |  |  | DAT |  |  | PAT |  |
| Asteraceae | *Verbesina palmeri* | TIB | ANG |  | LOR |  |  |  |  |  |  |  |  |  |  |
| Asteraceae | *Xanthisma incisifolium* |  |  | EST | LOR |  |  |  |  |  |  |  |  |  |  |
| Asteraceae | *Xanthisma scabrellum* | TIB | ANG |  |  |  |  |  |  |  |  |  |  |  |  |
| Asteraceae | *Xylorhiza frutescens* |  | ANG |  |  |  |  |  |  |  |  |  |  |  |  |
| Asteraceae | *Xylothamnia diffusa* | TIB | ANG |  | LOR |  |  |  | ALC |  |  |  |  |  |  |
| Bataceae | *Batis maritima* | TIB | ANG |  |  |  |  |  | ALC |  |  |  | RAS |  |  |
| Boraginaceae | *Bourreria sonorae* | TIB |  |  |  |  |  |  |  |  |  |  |  |  |  |
| Boraginaceae | *Cryptantha maritima* | TIB | ANG | EST | LOR | ANI |  |  |  | PAR |  |  |  |  |  |
| Boraginaceae | *Johnstonella angelica* | TIB | ANG |  |  |  |  |  |  |  |  |  |  |  |  |
| Boraginaceae | *Johnstonella angustifolia* | TIB | ANG | EST |  |  |  |  |  |  |  |  |  |  |  |
| Boraginaceae | *Johnstonella fastigiata* | TIB | ANG | EST | LOR | ANI |  |  |  |  | DAT |  |  |  |  |
| Boraginaceae | *Johnstonella grayi* var. *cryptochaeta* |  |  |  |  |  |  |  |  |  |  | SAL (?) |  |  |  |
| Boraginaceae | *Pectocarya linearis* subsp. *ferocula* |  | ANG |  |  |  |  |  |  |  |  |  |  |  |  |
| Boraginaceae | *Pectocarya recurvata* | TIB |  |  |  |  |  |  |  |  |  |  |  |  |  |
| Boraginaceae | *Plagiobothyrs jonesii* |  | ANG |  |  |  |  |  |  |  |  |  |  |  |  |
| Brassicaceae | *Caulanthus lasiophyllus* |  | ANG |  |  |  |  |  |  |  |  |  |  |  |  |
| Brassicaceae | *Descurainia pinnata* | TIB |  |  |  |  |  |  |  |  | DAT |  |  |  |  |
| Brassicaceae | *Lyrocarpa coulteri* | TIB |  |  |  |  |  |  |  |  | DAT |  |  |  |  |
| Brassicaceae | *Lyrocarpa linearifolia* |  | ANG | EST |  |  |  |  |  |  |  |  |  |  |  |
| Brassicaceae | *Sibara pectinata* |  | ANG |  |  |  |  |  |  |  |  |  |  |  |  |
| Brassicaceae | *Tomostima cuneifolia* | TIB |  | EST |  |  |  |  |  |  |  |  |  |  |  |
| Brassicaceae | *Tomostima cuneifolia* var. *integrifolia* |  | ANG |  |  |  |  |  |  |  |  |  |  |  |  |
| Burseraceae | *Bursera fagaroides* var. *elongata* | TIB |  |  |  |  |  |  |  |  |  |  |  |  |  |
| Burseraceae | *Bursera hindsiana* | TIB | ANG | EST |  |  |  |  |  |  | DAT |  |  |  | CHO |
| Burseraceae | *Bursera laxiflora* | TIB |  |  |  |  |  |  |  |  |  |  |  |  |  |
| Burseraceae | *Bursera microphylla* | TIB | ANG | EST | LOR |  | NOL |  | ALC |  | DAT |  |  |  |  |
| Cactaceae | *Carnegiea gigantea* | TIB |  |  |  |  |  |  | ALC |  |  |  |  | PAT | CHO |
| Cactaceae | *Cochemiea setispina* |  | ANG |  |  |  |  |  |  |  |  |  |  |  |  |
| Cactaceae | *Cylindropuntia alcahes* var. *alcahes* |  | ANG | EST | LOR | ANI |  | MAR |  | PAR |  | SAL | RAS |  |  |
| Cactaceae | *Cylindropuntia bigelovii* | TIB | ANG | EST | LOR | ANI |  |  | ALC |  | DAT | SAL |  |  |  |
| Cactaceae | *Cylindropuntia cholla* |  |  |  | LOR | ANI |  | MAR |  |  |  |  |  |  |  |
| Cactaceae | *Cylindropuntia fulgida* | TIB | ANG |  | LOR |  | NOL |  | ALC | PAR |  | SAL | RAS | PAT | CHO |
| Cactaceae | *Cylindropuntia leptocaulis* | TIB |  |  |  |  |  |  |  |  | DAT |  |  |  |  |
| Cactaceae | *Cylindropuntia versicolor* | TIB |  |  |  |  |  |  |  |  | DAT |  |  |  |  |
| Cactaceae | *Echinocereus grandis* |  |  | EST | LOR | ANI |  |  |  |  |  |  |  |  |  |
| Cactaceae | *Echinocereus scopulorum* | TIB |  |  |  |  |  |  |  |  |  |  |  |  |  |
| Cactaceae | *Echinocereus websterianus* |  |  |  |  |  | NOL |  |  |  |  |  |  |  |  |
| Cactaceae | *Ferocactus emoryi* |  |  |  |  |  |  |  | ALC |  |  |  |  |  |  |
| Cactaceae | *Ferocactus johnstonianus* |  | ANG |  |  |  |  |  |  |  |  |  |  |  |  |
| Cactaceae | *Ferocactus tiburonensis* | TIB |  |  |  |  |  |  |  |  |  |  |  |  |  |
| Cactaceae | *Lophocereus schottii* var. *schottii* | TIB |  |  |  | ANI (?) |  |  | ALC | PAR | DAT |  | RAS | PAT | CHO |
| Cactaceae | *Mammillaria angelensis* |  | ANG |  |  |  |  |  |  |  |  |  |  |  |  |
| Cactaceae | *Mammillaria diocia* |  |  |  | LOR (?) | ANI (?) |  |  |  |  |  |  |  |  |  |
| Cactaceae | *Mammillaria estebanensis* |  |  | EST |  |  |  |  |  |  |  |  |  |  |  |
| Cactaceae | *Mammillaria grahamii* subsp. *sheldonii* | TIB |  |  |  |  |  |  | ALC |  |  |  |  |  |  |
| Cactaceae | *Mammillaria multidigitata* |  |  |  |  |  | NOL |  |  |  |  |  |  |  |  |
| Cactaceae | *Mammillaria* sp. |  |  |  |  |  |  |  |  |  | DAT |  |  |  | CHO |
| Cactaceae | *Mammillaria tayloriorum* |  |  |  |  |  | NOL |  |  |  |  |  |  |  |  |
| Cactaceae | *Opuntia bravoana* |  |  |  |  |  | NOL |  |  |  |  |  |  |  |  |
| Cactaceae | *Opuntia engelmannii* var. *engelmannii* |  |  |  |  |  |  |  | ALC |  |  |  |  |  |  |
| Cactaceae | *Pachycereus pringlei* | TIB | ANG | EST | LOR | ANI | NOL | MAR | ALC | PAR | DAT | SAL | RAS | PAT | CHO |
| Cactaceae | *Peniocereus striatus* | TIB |  |  |  |  |  |  |  |  | DAT | SAL |  |  |  |
| Cactaceae | *Stenocereus gummosus* | TIB | ANG | EST | LOR | ANI |  |  |  |  | DAT | SAL | RAS | PAT | CHO |
| Cactaceae | *Stenocereus thurberi* | TIB | ANG (obs) | EST |  | ANI | NOL |  | ALC |  | DAT |  |  |  | CHO |
| Campanulaceae | *Nemacladus glanduliferus* var. *orientalis* |  | ANG |  | LOR |  |  |  |  |  |  |  |  |  |  |
| Campanulaceae | *Nemacladus orientalis* |  |  | EST |  |  |  |  |  |  |  |  |  |  |  |
| Cannabaceae | *Celtis pallida* | TIB |  |  |  |  |  |  |  |  |  |  |  |  |  |
| Cannabaceae | *Celtis reticulata* | TIB |  |  |  |  |  |  |  |  |  |  |  |  |  |
| Capparaceae | *Atamisquea emarginata* | TIB | ANG (obs) | EST |  |  |  |  |  |  |  |  |  |  | CHO |
| Caryophyllaceae | *Achyronychia cooperi* | TIB | ANG |  | LOR | ANI |  |  |  |  |  |  |  |  |  |
| Caryophyllaceae | *Drymaria holosteoides* var. *holosteoides* | TIB | ANG |  |  |  |  |  |  |  |  |  |  |  |  |
| Celastraceae | *Canotia holacantha* | TIB |  |  |  |  |  |  |  |  |  |  |  |  |  |
| Celastraceae | *Tricerma phyllanthoides* | TIB |  |  |  |  |  |  | ALC |  |  |  |  |  |  |
| Cleomaceae | *Cleome tenuis* | TIB |  |  |  |  |  |  |  |  |  |  |  |  |  |
| Cochlospermaceae | *Amoreuxia palmatifida* | TIB |  |  |  |  |  |  |  |  |  |  |  |  |  |
| Combretaceae | *Laguncularia racemosa* | TIB |  |  |  |  |  |  |  |  |  |  |  |  |  |
| Convolvulaceae | *Cressa truxillensis* | TIB | ANG |  | LOR |  |  |  | ALC |  |  | SAL | RAS |  |  |
| Convolvulaceae | *Cuscuta americana* | TIB |  |  |  |  |  |  |  |  |  |  |  |  |  |
| Convolvulaceae | *Cuscuta corymbosa* var. *grandiflora* |  | ANG | EST |  |  | NOL |  |  | PAR |  |  |  |  |  |
| Convolvulaceae | *Cuscuta desmouliniana* | TIB |  |  |  |  |  |  |  |  | DAT |  |  |  |  |
| Convolvulaceae | *Cuscuta leptantha* | TIB | ANG |  |  |  |  |  |  |  |  |  |  |  |  |
| Convolvulaceae | *Evolvulus alsinoides* | TIB |  |  |  |  |  |  |  |  |  |  |  |  |  |
| Convolvulaceae | *Ipomoea hederacea* | TIB |  |  |  |  |  |  |  |  |  |  |  |  |  |
| Convolvulaceae | *Ipomoea terniflora* var. *leptoloma* | TIB |  |  |  |  |  |  |  |  |  |  |  |  |  |
| Convolvulaceae | *Jacquemontia abutiloides* | TIB |  |  |  |  |  |  |  |  |  |  |  |  |  |
| Convolvulaceae | *Jacquemontia agrestis* | TIB |  |  |  |  |  |  |  |  |  |  |  |  |  |
| Cordiaceae | *Cordia parvifolia* | TIB |  |  |  |  |  |  |  |  |  |  |  |  |  |
| Crassulaceae | *Crassula connata* |  | ANG |  |  |  |  |  |  |  |  |  |  |  |  |
| Crassulaceae | *Dudleya arizonica* |  | ANG |  |  |  |  |  |  |  |  |  |  |  |  |
| Crossosomataceae | *Crossosoma bigelovii* | TIB |  |  |  |  |  |  |  |  |  |  |  |  |  |
| Cucurbitaceae | *Tumamoca macdougalii* | TIB |  |  |  |  |  |  |  |  |  |  |  |  |  |
| Cucurbitaceae | *Vaseyanthus insularis* | TIB | ANG | EST | LOR | ANI | NOL | MAR |  | PAR |  |  |  |  |  |
| Cymodaceae | *Halodule wrightii* | TIB |  |  |  |  |  |  |  |  |  |  |  |  |  |
| Cyperaceae | *Cyperus elegans* | TIB |  |  |  |  | NOL |  |  |  |  |  |  |  |  |
| Cyperaceae | *Cyperus* *squarrosus* |  |  |  |  |  | NOL | MAR |  |  |  |  |  |  |  |
| Cyperaceae | *Eleocharis geniculata* | TIB |  |  |  |  |  |  |  |  |  |  |  |  |  |
| Ehretiaceae | *Tiquilia canescens* | TIB |  |  |  |  |  |  |  |  | DAT |  |  |  |  |
| Ehretiaceae | *Tiquilia palmeri* | TIB | ANG |  |  |  |  |  |  |  |  |  |  |  |  |
| Ephedraceae | *Ephedra aspera* | TIB |  | EST | LOR |  |  |  |  |  |  |  |  |  |  |
| Euphorbiaceae | *Acalypha californica* | TIB |  | EST |  |  |  |  |  |  | DAT |  |  |  |  |
| Euphorbiaceae | *Argythamnia brandegeei* |  | ANG |  |  |  |  |  |  |  |  |  |  |  |  |
| Euphorbiaceae | *Argythamnia lanceolata* | TIB | ANG | EST |  |  |  |  |  |  | DAT |  |  |  |  |
| Euphorbiaceae | *Argythamnia neomexicana* | TIB | ANG |  | LOR | ANI |  |  |  |  | DAT |  |  |  |  |
| Euphorbiaceae | *Argythamnia serrata* |  |  | EST |  |  |  |  |  |  |  |  |  |  |  |
| Euphorbiaceae | *Bernardia viridis* |  |  |  |  |  | NOL |  |  |  |  |  |  |  |  |
| Euphorbiaceae | *Cnidosculus palmeri* | TIB |  |  |  |  |  |  |  |  | DAT |  |  |  |  |
| Euphorbiaceae | *Croton californicus* | TIB |  |  |  |  |  |  |  |  |  |  |  |  |  |
| Euphorbiaceae | *Croton magdalenae* | TIB |  |  |  |  |  |  |  |  |  |  |  |  |  |
| Euphorbiaceae | *Croton sonorae* | TIB |  |  |  |  |  |  |  |  |  |  |  |  |  |
| Euphorbiaceae | *Euphorbia abramsiana* | TIB |  |  |  |  |  |  |  |  |  |  |  |  |  |
| Euphorbiaceae | *Euphorbia arizonica* | TIB |  |  |  |  |  |  |  |  |  |  |  |  |  |
| Euphorbiaceae | *Euphorbia eriantha* | TIB | ANG |  |  |  |  |  |  |  | DAT |  |  |  |  |
| Euphorbiaceae | *Euphorbia florida* | TIB |  |  |  |  |  |  |  |  |  |  |  |  |  |
| Euphorbiaceae | *Euphorbia leucophylla* subsp. *comcaacorum* | TIB |  |  |  |  |  |  |  |  |  |  |  |  |  |
| Euphorbiaceae | *Euphorbia lomelii* |  |  |  |  |  | NOL |  |  |  |  |  |  |  |  |
| Euphorbiaceae | *Euphorbia magdalenae* | TIB |  |  |  |  | NOL |  |  |  |  |  |  |  |  |
| Euphorbiaceae | *Euphorbia misera* | TIB | ANG | EST |  |  |  |  |  |  | DAT |  |  |  |  |
| Euphorbiaceae | *Euphorbia pediculifera* var. *pediculifera* | TIB | ANG | EST | LOR | ANI |  |  |  |  | DAT |  |  |  |  |
| Euphorbiaceae | *Euphorbia petrina* | TIB |  | EST |  |  |  | MAR | ALC |  |  |  |  |  |  |
| Euphorbiaceae | *Euphorbia polycarpa* | TIB | ANG | EST |  |  |  |  |  | PAR (?) | DAT | SAL |  |  |  |
| Euphorbiaceae | *Euphorbia prostrata* | TIB |  |  |  |  |  |  |  |  |  |  |  |  |  |
| Euphorbiaceae | *Euphorbia setiloba* | TIB | ANG | EST |  |  |  |  |  |  |  |  |  |  |  |
| Euphorbiaceae | *Euphorbia tomentulosa* | TIB |  |  |  |  |  |  |  |  |  |  |  |  |  |
| Euphorbiaceae | *Euphorbia xanti* | TIB |  |  |  |  |  |  |  |  |  |  |  |  |  |
| Euphorbiaceae | *Jatropha cinerea* | TIB |  |  |  |  |  |  |  |  |  |  |  |  |  |
| Euphorbiaceae | *Jatropha cuneata* | TIB | ANG | EST | LOR | ANI | NOL |  |  |  | DAT |  |  |  |  |
| Euphorbiaceae | *Pleradenophora bilocularis* | TIB |  |  |  |  |  |  |  |  |  |  |  |  |  |
| Euphorbiaceae | *Tragia jonesii* | TIB |  |  |  |  |  |  |  |  | DAT |  |  |  |  |
| Fabaceae | *Acmispon maritimus* var. *brevivexillus* | TIB | ANG |  | LOR | ANI |  |  |  |  |  |  |  |  |  |
| Fabaceae | *Acmispon rigidus* |  | ANG |  |  |  |  |  |  |  |  |  |  |  |  |
| Fabaceae | *Acmispon strigosus* | TIB | ANG | EST | LOR | ANI |  |  |  |  |  |  |  |  |  |
| Fabaceae | *Astragalus insularis* var. *harwoodii* |  | ANG |  | LOR | ANI |  |  |  |  |  |  |  |  |  |
| Fabaceae | *Astragalus nuttallianus* var. *cedrosensis* |  | ANG |  | LOR | ANI |  |  |  |  |  |  |  |  |  |
| Fabaceae | *Calliandra californica* | TIB |  |  |  |  |  |  |  |  | DAT |  |  |  |  |
| Fabaceae | *Calliandra eriophylla* var. *eriophylla* | TIB |  |  |  |  |  |  |  |  |  |  |  |  |  |
| Fabaceae | *Coursetia glandulosa* | TIB |  |  |  |  |  |  |  |  |  |  |  |  |  |
| Fabaceae | *Dalea bicolor* var. *orcuttiana* | TIB |  |  |  |  |  |  |  |  |  |  |  |  |  |
| Fabaceae | *Dalea mollis* | TIB | ANG |  |  |  |  |  | ALC |  | DAT |  |  |  |  |
| Fabaceae | *Desmanthus covillei* | TIB |  |  |  |  |  |  |  |  |  |  |  |  |  |
| Fabaceae | *Desmanthus fruticosus* | TIB | ANG | EST |  |  |  |  |  |  | DAT |  |  |  |  |
| Fabaceae | *Desmodium procumbens* | TIB |  |  |  |  |  |  |  |  |  |  |  |  |  |
| Fabaceae | *Ebenopsis confinis* | TIB |  |  |  |  |  |  |  |  | DAT |  |  |  |  |
| Fabaceae | *Errazurizia megacarpa* | TIB | ANG |  |  |  |  |  |  |  |  |  |  |  |  |
| Fabaceae | *Hoffmannseggia intricata* | TIB | ANG | EST |  |  |  |  |  |  | DAT |  |  |  |  |
| Fabaceae | *Hoffmannseggia microphylla* |  | ANG |  |  |  |  |  |  |  |  |  |  |  |  |
| Fabaceae | *Lupinus arizonicus* | TIB | ANG | EST | LOR | ANI |  |  |  |  |  |  |  |  |  |
| Fabaceae | *Lysiloma divaricatum* | TIB |  |  |  |  |  |  |  |  |  |  |  |  |  |
| Fabaceae | *Marina evanescens* |  |  | EST |  |  |  |  |  |  |  |  |  |  |  |
| Fabaceae | *Marina parryi* | TIB | ANG | EST |  |  |  |  |  |  | DAT |  |  |  |  |
| Fabaceae | *Marina vetula* | TIB |  |  |  |  |  |  |  |  |  |  |  |  |  |
| Fabaceae | *Mariosousa heterophylla* | TIB |  |  |  |  | NOL |  |  |  |  |  |  |  |  |
| Fabaceae | *Mimosa distachya* var. *laxiflora* | TIB |  |  |  |  |  |  |  |  |  |  |  |  |  |
| Fabaceae | *Olneya tesota* | TIB | ANG | EST |  |  |  |  | ALC |  | DAT |  |  |  | CHO |
| Fabaceae | *Parkinsonia florida* subsp. *florida* | TIB |  |  |  |  |  |  |  |  |  |  |  |  |  |
| Fabaceae | *Parkinsonia microphylla* | TIB | ANG | EST |  |  |  |  |  |  |  |  |  |  |  |
| Fabaceae | *Phaseolus acutifolius* | TIB |  |  |  |  |  |  |  |  |  |  |  |  |  |
| Fabaceae | *Phaseolus filiformis* | TIB | ANG | EST | LOR | ANI |  |  |  | PAR | DAT |  |  |  |  |
| Fabaceae | *Pithecellobium dulce** | TIB |  |  |  |  |  |  |  |  |  |  |  |  |  |
| Fabaceae | *Prosopis glandulosa* var. *torreyana* | TIB | ANG | EST |  |  |  |  | ALC |  | DAT |  |  |  |  |
| Fabaceae | *Psorothamnus emoryi* var. *emoryi* | TIB | ANG |  | LOR |  |  |  |  |  |  |  |  |  |  |
| Fabaceae | *Psorothamnus spinosus* |  | ANG |  |  |  |  |  |  |  |  |  |  |  |  |
| Fabaceae | *Rhynchosia precatoria* | TIB |  |  |  |  |  |  |  |  |  |  |  |  |  |
| Fabaceae | *Senegalia greggii* | TIB | ANG |  |  |  |  |  |  |  |  |  |  |  |  |
| Fabaceae | *Senna confinis* | TIB | ANG | EST |  |  |  |  |  |  |  |  |  |  |  |
| Fabaceae | *Senna covesii* | TIB |  |  |  |  |  |  |  |  | DAT |  |  |  |  |
| Fabaceae | *Sphinctospermum constrictum* | TIB |  |  |  |  |  |  |  |  |  |  |  |  |  |
| Fabaceae | *Tephrosia palmeri* | TIB |  |  |  |  |  |  |  |  |  |  |  |  |  |
| Fabaceae | *Tephrosia tenella* | TIB |  |  |  |  |  |  |  |  |  |  |  |  |  |
| Fabaceae | *Zapoteca formosa* subsp. *rosei* | TIB |  |  |  |  |  |  |  |  |  |  |  |  |  |
| Fouquieriaceae | *Fouquieria columnaris* |  | ANG |  |  |  |  |  |  |  |  |  |  |  |  |
| Fouquieriaceae | *Fouquieria diguetii* |  | ANG |  | LOR |  | NOL |  |  |  |  |  |  |  |  |
| Fouquieriaceae | *Fouquieria splendens* subsp. *splendens* | TIB | ANG | EST |  |  |  |  |  |  | DAT |  |  |  |  |
| Frankeniaceae | *Frankenia palmeri* | TIB | ANG |  |  |  |  |  |  |  |  |  |  |  |  |
| Heliotropiaceae | *Heliotropium curassavicum* | TIB | ANG |  | LOR |  |  |  | ALC |  |  |  |  |  |  |
| Heliotropiaceae | *Heliotropium fruticosum* | TIB |  |  |  |  |  |  |  |  |  |  |  |  |  |
| Heliotropiaceae | *Tournefortia hartwegiana* | TIB |  |  |  |  |  |  |  |  |  |  |  |  |  |
| Hydrophyllaceae | *Eucrypta micrantha* | TIB |  |  |  |  |  |  |  |  |  |  |  |  |  |
| Hydrophyllaceae | *Phacelia affinis* | TIB |  |  |  |  |  |  |  |  |  |  |  |  |  |
| Hydrophyllaceae | *Phacelia crenulata* | TIB |  |  |  |  |  |  |  |  |  |  |  |  |  |
| Hydrophyllaceae | *Phacelia pauciflora* |  | ANG |  |  |  |  |  |  |  |  |  |  |  |  |
| Hydrophyllaceae | *Phacelia pedicellata* | TIB | ANG | EST |  |  |  |  |  |  | DAT |  |  |  |  |
| Hydrophyllaceae | *Pholistoma racemosum* |  | ANG |  |  |  |  |  |  |  |  |  |  |  |  |
| Koeberlineaceae | *Koeberlinia spinosa* var. *tenuispina* | TIB |  |  |  |  |  |  |  |  |  |  |  |  |  |
| Krameriaceae | *Krameria bicolor* | TIB |  |  |  |  |  |  |  |  |  |  |  |  |  |
| Krameriaceae | *Krameria erecta* | TIB |  |  |  |  |  |  |  |  |  |  |  |  |  |
| Lamiaceae | *Hyptis albida* | TIB | ANG | EST | LOR |  |  |  |  |  | DAT |  |  |  |  |
| Lamiaceae | *Monardella lagunensis* |  | ANG |  |  |  |  |  |  |  |  |  |  |  |  |
| Lamiaceae | *Salvia similis* |  |  |  |  |  | NOL |  |  |  |  |  |  |  |  |
| Loasaceae | *Eucnide cordata* |  | ANG | EST | LOR | ANI |  |  |  |  |  |  |  |  |  |
| Loasaceae | *Eucnide rupestris* | TIB | ANG | EST |  |  | NOL |  |  | PAR | DAT | SAL |  |  | CHO |
| Loasaceae | *Mentzelia adhaerans* | TIB | ANG | EST | LOR | ANI |  | MAR | ALC |  | DAT |  |  |  |  |
| Loasaceae | *Mentzelia hirsutissima* | TIB | ANG |  |  |  |  |  |  |  |  |  |  |  |  |
| Loasaceae | *Petalonyx linearis* | TIB | ANG | EST | LOR |  |  | MAR |  |  |  |  |  |  |  |
| Malpighiaceae | *Callaeum macropterum* | TIB |  |  |  |  |  |  |  |  |  |  |  |  |  |
| Malpighiaceae | *Cottsia californica* | TIB |  |  |  |  |  |  |  |  | DAT |  |  |  |  |
| Malpighiaceae | *Cottsia gracilis* | TIB |  | EST |  |  |  |  |  |  | DAT |  |  |  |  |
| Malpighiaceae | *Echinopterys eglandulosa* | TIB |  |  |  |  |  |  |  |  |  |  |  |  |  |
| Malpighiaceae | *Galphimia angustifolia* | TIB |  |  |  |  | NOL |  |  |  |  |  |  |  |  |
| Malvaceae | *Abutilon californicum* | TIB |  |  |  |  |  |  |  |  |  |  |  |  |  |
| Malvaceae | *Abutilon incanum* | TIB |  |  |  |  |  |  |  |  | DAT |  |  |  |  |
| Malvaceae | *Abutilon palmeri* | TIB | ANG |  |  |  |  | MAR |  |  |  |  |  |  |  |
| Malvaceae | *Ayenia compacta* |  | ANG |  |  |  |  |  |  |  |  |  |  |  |  |
| Malvaceae | *Ayenia filiformis* | TIB |  | EST |  |  |  |  |  |  |  |  |  |  |  |
| Malvaceae | *Gossypium davidsonii* |  |  |  |  |  | NOL |  |  |  |  |  |  |  |  |
| Malvaceae | *Herissantia crispa* | TIB |  |  |  |  |  |  |  |  | DAT |  |  |  |  |
| Malvaceae | *Hibiscus biseptus* | TIB |  |  |  |  |  |  |  |  |  |  |  |  |  |
| Malvaceae | *Hibiscus denudatus* | TIB | ANG | EST |  |  |  |  |  |  | DAT |  |  |  |  |
| Malvaceae | *Horsfordia alata* | TIB |  |  |  |  |  |  |  |  |  |  |  |  |  |
| Malvaceae | *Horsfordia newberryi* | TIB | ANG | EST |  |  |  |  |  |  | DAT |  |  |  |  |
| Malvaceae | *Melochia tomentosa* | TIB |  |  |  |  |  |  |  |  | DAT |  |  |  |  |
| Malvaceae | *Sida abutifolia* | TIB |  |  |  |  |  |  |  |  |  |  |  |  |  |
| Malvaceae | *Sphaeralcea ambigua* var. *ambigua* | TIB |  |  |  |  |  |  |  |  | DAT |  |  |  |  |
| Malvaceae | *Sphaeralcea ambigua* var. *versicolor* |  | ANG | EST |  | ANI |  |  |  |  |  |  |  |  |  |
| Malvaceae | *Sphaeralcea coulteri* |  |  |  |  |  |  |  | ALC |  |  |  |  |  |  |
| Malvaceae | *Sphaeralcea hainesii* |  |  |  | LOR |  |  | MAR |  |  |  |  |  |  |  |
| Malvaceae | *Waltheria indica* | TIB |  |  |  |  |  |  |  |  |  |  |  |  |  |
| Martyniaceae | *Proboscidea altheifolia* | TIB |  |  |  |  |  |  |  |  |  |  |  |  |  |
| Molluginaceae | *Mollugo cerviana** | TIB |  |  |  |  |  |  |  |  |  |  |  |  |  |
| Molluginaceae | *Mollugo verticillata* |  |  |  |  |  | NOL |  |  |  |  |  |  |  |  |
| Montiaceae | *Calandrinia maritima* |  | ANG |  |  |  |  |  |  |  |  |  |  |  |  |
| Moraceae | *Ficus palmeri* | TIB |  | EST | LOR |  | NOL | MAR | ALC |  | DAT |  |  |  | CHO |
| Myrtaceae | *Eucalyptus camaldulensis** | TIB |  |  |  |  |  |  |  |  |  |  |  |  |  |
| Namaceae | *Nama hispida* | TIB |  |  |  |  |  |  |  |  |  |  |  |  |  |
| Nyctaginaceae | *Abronia maritima* subsp. *maritima* | TIB | ANG |  | LOR | ANI |  |  | ALC |  |  | SAL |  |  |  |
| Nyctaginaceae | *Allionia incarnata* | TIB | ANG |  |  | ANI |  |  |  |  | DAT |  |  |  |  |
| Nyctaginaceae | *Boerhavia triquetra* | TIB | ANG | EST |  |  | NOL |  |  |  | DAT |  |  |  |  |
| Nyctaginaceae | *Boerhavia xanti* | TIB |  |  |  |  |  |  |  |  |  |  |  |  |  |
| Nyctaginaceae | *Commicarpus scandens* | TIB |  |  |  |  |  |  |  |  |  |  |  |  |  |
| Nyctaginaceae | *Mirabilis laevis* var. *crassifolia* |  |  | EST |  |  |  |  |  |  |  |  |  |  |  |
| Nyctaginaceae | *Mirabilis tenuiloba* | TIB | ANG | EST | LOR (obs) | ANI |  |  |  |  |  |  |  |  |  |
| Oleaceae | *Forestiera phillyreoides* | TIB |  |  |  |  |  |  |  |  |  |  |  |  |  |
| Oleaceae | *Fraxinus gooddingii* | TIB |  |  |  |  |  |  |  |  |  |  |  |  |  |
| Oleaceae | *Menodora scabra* | TIB |  |  |  |  |  |  |  |  |  |  |  |  |  |
| Onagraceae | *Chylismia cardiophylla* subsp. *cardiophylla* |  | ANG | EST | LOR |  |  | MAR |  |  |  |  |  |  |  |
| Onagraceae | *Chylismia cardiophylla* subsp. *cedrosensis* | TIB |  |  |  |  |  |  |  |  |  |  |  |  |  |
| Onagraceae | *Eremothera chamaenerioides* | TIB | ANG |  |  |  |  |  |  |  |  |  |  |  |  |
| Onagraceae | *Eulobus californicus* | TIB | ANG |  |  |  |  |  |  |  |  |  |  |  |  |
| Onagraceae | *Eulobus crassifolia* |  |  |  | LOR |  |  |  |  |  |  |  |  |  |  |
| Onagraceae | *Oenothera brandegeei* |  | ANG |  |  |  |  |  |  |  |  |  |  |  |  |
| Onagraceae | *Oenothera primiveris* | TIB |  |  |  |  |  |  |  |  |  |  |  |  |  |
| Orobanchaceae | *Orobanche cooperi* | TIB | ANG |  |  |  |  |  |  |  |  |  |  |  |  |
| Papaveraceae | *Argemone subintegrifolia* |  | ANG | EST |  |  |  |  |  |  |  |  |  |  |  |
| Papaveraceae | *Eschscholzia minutiflora* |  | ANG |  |  |  |  |  |  |  |  |  |  |  |  |
| Papaveraceae | *Eschscholzia parishii* |  | ANG |  |  |  |  |  |  |  |  |  |  |  |  |
| Passifloraceae | *Passiflora arida* | TIB |  | EST | LOR |  |  |  |  |  |  |  |  |  |  |
| Passifloraceae | *Passiflora palmeri* | TIB | ANG | EST | LOR |  |  |  |  |  | DAT |  |  |  |  |
| Petiveriaceae | *Rivina humilis* | TIB |  |  |  |  |  |  |  |  |  |  |  |  |  |
| Phyllanthaceae | *Andrachne microphylla* | TIB | ANG |  |  |  |  |  |  |  |  |  |  |  |  |
| Plantaginaceae | *Castilleja lanata* |  | ANG |  |  |  |  |  |  |  |  |  |  |  |  |
| Plantaginaceae | *Gambelia juncea* | TIB | ANG | EST | LOR |  | NOL |  |  |  |  |  |  |  |  |
| Plantaginaceae | *Mohavea confertiflora* |  | ANG |  |  |  |  |  |  |  |  |  |  |  |  |
| Plantaginaceae | *Nuttallanthus texanus* | TIB |  |  |  |  |  |  |  |  |  |  |  |  |  |
| Plantaginaceae | *Penstemon angelicus* |  | ANG |  |  |  |  |  |  |  |  |  |  |  |  |
| Plantaginaceae | *Plantago ovata* var. *fastigiata* | TIB | ANG |  |  | ANI |  |  |  |  |  |  |  |  |  |
| Plantaginaceae | *Pseudorontium cyathiferum* | TIB | ANG | EST |  |  | NOL |  |  |  | DAT |  |  |  | CHO |
| Plantaginaceae | *Sairocarpus watsonii* | TIB | ANG |  | LOR | ANI |  |  |  |  | DAT |  |  |  |  |
| Plantaginaceae | *Stemodia durantifolia* | TIB |  |  |  |  |  |  |  |  |  |  |  |  |  |
| Plumbaginaceae | *Plumbago zeylanica* | TIB |  |  |  |  |  |  |  |  |  |  |  |  |  |
| Poaceae | *Aristida adscensionis* | TIB | ANG | EST | LOR | ANI | NOL | MAR |  |  | DAT |  |  |  |  |
| Poaceae | *Aristida californica* var. *californica* | TIB | ANG |  | LOR |  |  |  |  |  |  |  |  |  |  |
| Poaceae | *Aristida californica* var. *glabrata* | TIB |  |  |  |  |  |  |  |  |  |  |  |  |  |
| Poaceae | *Aristida divaricata* |  |  |  |  |  | NOL |  |  |  |  |  |  |  |  |
| Poaceae | *Aristida purpurea* var. *nealleyi* |  | ANG |  | LOR (obs) |  |  |  |  |  |  |  |  |  |  |
| Poaceae | *Aristida ternipes* var. *ternipes* | TIB |  |  |  |  | NOL |  |  |  |  |  |  |  |  |
| Poaceae | *Arundo donax** | TIB |  |  |  |  |  |  |  |  |  |  |  |  |  |
| Poaceae | *Bothriochloa barbinodis* |  | ANG | EST |  |  | NOL |  |  |  |  |  |  |  |  |
| Poaceae | *Bouteloua aristidoides* | TIB |  | EST |  |  | NOL |  | ALC |  | DAT |  |  |  | CHO |
| Poaceae | *Bouteloua barbata* var. *barbata* | TIB | ANG | EST |  |  |  | MAR | ALC | PAR |  |  |  | PAT |  |
| Poaceae | *Bouteloua diversispicula* | TIB |  |  |  |  |  |  |  |  |  |  |  |  |  |
| Poaceae | *Cenchrus ciliaris** | TIB |  |  |  |  |  | MAR | ALC |  |  |  |  |  |  |
| Poaceae | *Cenchrus echinatus** | TIB |  |  |  |  |  |  |  |  |  |  |  |  |  |
| Poaceae | *Cenchrus palmeri* | TIB | ANG | EST | LOR |  | NOL |  |  |  |  |  |  |  |  |
| Poaceae | *Chloris virgata* | TIB |  |  |  |  |  |  |  |  |  |  |  |  |  |
| Poaceae | *Digitaria californica* var. *californica* | TIB |  | EST |  |  | NOL | MAR |  |  | DAT |  |  |  | CHO |
| Poaceae | *Disakisperma dubium* | TIB |  |  |  |  |  |  |  |  |  |  |  |  |  |
| Poaceae | *Distichlis littoralis* | TIB | ANG |  | LOR |  |  |  | ALC |  |  |  | RAS |  |  |
| Poaceae | *Distichlis palmeri* |  | ANG |  |  |  |  |  |  |  |  |  |  |  |  |
| Poaceae | *Distichlis spicata* | TIB |  |  |  |  |  |  |  |  |  |  |  |  |  |
| Poaceae | *Enneapogon desvauxii* | TIB |  |  |  |  |  |  |  |  |  |  |  |  |  |
| Poaceae | *Enteropogon chlorideus* |  |  | EST |  |  |  |  |  |  |  |  |  |  |  |
| Poaceae | *Eragrostis cilianensis* | TIB |  |  |  |  |  |  |  |  |  |  |  |  |  |
| Poaceae | *Eragrostis pectinacea* var. *pectinacea* |  |  |  |  |  | NOL |  |  |  |  |  |  |  |  |
| Poaceae | *Festuca octoflora* |  | ANG |  |  |  |  |  |  |  |  |  |  |  |  |
| Poaceae | *Heteropogon contortus* | TIB | ANG | EST | LOR |  | NOL |  |  |  |  |  |  |  |  |
| Poaceae | *Lasiacis ruscifolia* var. *ruscifolia* | TIB |  |  |  |  |  |  |  |  |  |  |  |  |  |
| Poaceae | *Leptochloa crinita* | TIB |  |  |  |  | NOL |  |  |  |  |  |  |  |  |
| Poaceae | *Leptochloa panicea* subsp. *brachiata* | TIB |  | EST |  |  | NOL |  |  |  |  |  |  |  |  |
| Poaceae | *Melica frutescens* |  | ANG |  |  |  |  |  |  |  |  |  |  |  |  |
| Poaceae | *Muhlenbergia microsperma* | TIB | ANG | EST | LOR | ANI | NOL | MAR |  |  |  |  |  |  |  |
| Poaceae | *Munroa pulchella* | TIB | ANG | EST | LOR |  |  |  |  |  | DAT |  |  |  |  |
| Poaceae | *Panicum hirticaule* var. *hirticaule* | TIB |  | EST |  |  |  |  |  |  |  |  |  |  | CHO |
| Poaceae | *Pappostipa speciosa* |  | ANG |  |  |  |  |  |  |  |  |  |  |  |  |
| Poaceae | *Phragmites australis* subsp. *berlandieri* | TIB |  |  |  |  |  |  |  |  |  |  |  |  |  |
| Poaceae | *Setaria liebmannii* | TIB |  |  |  |  | NOL |  |  |  |  |  |  |  |  |
| Poaceae | *Setaria macrostachya* | TIB |  | EST |  |  | NOL |  |  |  | DAT |  |  |  | CHO |
| Poaceae | *Sporobolus coromandelianus* | TIB |  |  |  |  |  |  |  |  |  |  |  |  |  |
| Poaceae | *Sporobolus cryptandrus* | TIB |  |  |  |  |  |  |  |  |  |  |  |  |  |
| Poaceae | *Sporobolus virginicus* | TIB |  |  |  |  |  |  | ALC |  |  |  |  |  |  |
| Poaceae | *Tridentopsis mutica* | TIB |  |  |  |  |  |  |  |  | DAT |  |  |  |  |
| Poaceae | *Urochloa arizonica* | TIB |  | EST |  |  |  |  |  |  | DAT |  |  |  | CHO |
| Poaceae | *Urochloa fusca* | TIB |  | EST |  |  |  |  |  |  |  |  |  |  |  |
| Polemoniaceae | *Bryantiella palmeri* |  | ANG |  |  |  |  |  |  |  |  |  |  |  |  |
| Polemoniaceae | *Gilia stellata* |  | ANG |  |  |  |  |  |  |  |  |  |  |  |  |
| Polemoniaceae | *Linanthus pungens* |  | ANG |  |  |  |  |  |  |  |  |  |  |  |  |
| Polygonaceae | *Eriogonum angelense* |  | ANG |  |  |  |  |  |  |  |  |  |  |  |  |
| Polygonaceae | *Eriogonum austrinum* |  | ANG |  |  |  |  |  |  |  |  |  |  |  |  |
| Polygonaceae | *Eriogonum elongatum* |  | ANG |  |  |  |  |  |  |  |  |  |  |  |  |
| Polygonaceae | *Eriogonum fasciculatum* var. *flavoviride* |  | ANG |  |  |  |  |  |  |  |  |  |  |  |  |
| Polygonaceae | *Eriogonum fasciculatum* var. *polifolium* |  | ANG |  |  |  |  |  |  |  |  |  |  |  |  |
| Polygonaceae | *Eriogonum inflatum* | TIB | ANG | EST | LOR |  |  |  |  |  |  |  |  |  |  |
| Polygonaceae | *Eriogonum orcuttianum* |  | ANG |  |  |  |  |  |  |  |  |  |  |  |  |
| Polygonaceae | *Eriogonum thomasii* |  | ANG |  |  |  |  |  |  |  |  |  |  |  |  |
| Polygonaceae | *Eriogonum wrightii* var. *linearifolium* |  | ANG |  |  |  |  |  |  |  |  |  |  |  |  |
| Portulacaceae | *Portulaca californica* |  |  |  |  | ANI |  |  |  |  |  |  |  |  |  |
| Portulacaceae | *Portulaca halimoides* | TIB |  |  |  |  |  |  |  |  |  |  |  |  |  |
| Portulacaceae | *Portulaca oleracea* | TIB |  |  |  |  |  |  |  |  |  |  |  |  |  |
| Portulacaceae | *Portulaca umbraticola* subsp. *lanceolata* | TIB |  |  |  |  |  |  |  |  |  |  |  |  |  |
| Pteridaceae | *Astrolepis sinuata* subsp. *sinuata* | TIB |  |  |  |  |  |  |  |  |  |  |  |  |  |
| Pteridaceae | *Cheilanthes brandegeii* |  | ANG |  |  |  |  |  |  |  |  |  |  |  |  |
| Pteridaceae | *Myriopteris wrightii* | TIB |  |  |  |  |  |  |  |  |  |  |  |  |  |
| Pteridaceae | *Notholaena californica* subsp. *californica* | TIB | ANG | EST |  |  |  |  |  |  |  |  |  |  |  |
| Pteridaceae | *Notholaena californica* subsp. *leucophylla* |  | ANG |  |  |  |  |  |  |  |  |  |  |  |  |
| Pteridaceae | *Notholaena lemmonii* var. *lemmonii* | TIB |  |  |  |  | NOL |  |  |  |  |  |  |  |  |
| Pteridaceae | *Notholaena standleyi* | TIB | ANG |  |  |  |  |  |  |  |  |  |  |  |  |
| Resedaceae | *Oligomeris linifolia* | TIB | ANG | EST | LOR |  |  |  |  |  | DAT | SAL |  |  |  |
| Rhamnaceae | *Colubrina viridis* | TIB | ANG | EST |  |  | NOL |  |  |  | DAT |  |  |  | CHO |
| Rhamnaceae | *Condalia globosa* var. *pubescens* | TIB | ANG | EST |  |  |  |  |  |  | DAT |  |  |  |  |
| Rhamnaceae | *Sageretia wrightii* | TIB |  |  |  |  |  |  |  |  |  |  |  |  |  |
| Rhamnaceae | *Sarcomphalus obtusifolius* var. *canescens* | TIB | ANG | EST |  |  |  |  |  |  | DAT |  |  |  |  |
| Rhizophoraceae | *Rhizophora mangle* | TIB |  |  |  |  |  |  |  |  |  |  | RAS |  |  |
| Rubiaceae | *Chiococca petrina* | TIB |  |  |  |  |  |  |  |  |  |  |  |  |  |
| Rubiaceae | *Galium proliferum* | TIB |  |  |  |  |  |  |  |  |  |  |  |  |  |
| Rubiaceae | *Galium stellatum* var. *eremicum* |  | ANG | EST | LOR |  |  |  |  |  |  |  |  |  |  |
| Rubiaceae | *Hedyotis brevipes* |  |  |  | LOR |  |  |  |  |  |  |  |  |  |  |
| Rubiaceae | *Randia thurberi* | TIB |  |  |  |  |  |  |  |  |  |  |  |  |  |
| Ruppiaceae | *Ruppia maritima* | TIB | ANG |  |  |  |  |  |  |  |  |  |  |  |  |
| Rutaceae | *Thamnosma montana* |  | ANG |  |  |  |  |  |  |  |  |  |  |  |  |
| Salicaceae | *Salix exigua* | TIB |  |  |  |  |  |  |  |  |  |  |  |  |  |
| Santalaceae | *Phoradendron brachystachyum* | TIB |  |  |  |  |  |  |  |  |  |  |  |  |  |
| Santalaceae | *Phoradendron californicum* | TIB | ANG |  |  |  |  |  |  |  |  |  |  |  |  |
| Sapindaceae | *Cardiospermum corindum* | TIB |  |  |  |  |  |  |  |  | DAT |  |  |  |  |
| Sapindaceae | *Dodonaea viscosa* | TIB | ANG |  |  |  |  |  |  |  |  |  |  |  |  |
| Sapotaceae | *Sideroxylon leucophyllum* | TIB | ANG | EST |  |  |  |  |  |  |  |  |  |  |  |
| Sapotaceae | *Sideroxylon occidentale* | TIB |  |  |  |  |  |  |  |  |  |  |  |  |  |
| Simaroubaceae | *Castela polyandra* | TIB |  |  |  |  |  |  |  |  |  |  |  |  |  |
| Simmondsiaceae | *Simmondsia chinensis* | TIB | ANG | EST |  |  | NOL |  |  |  | DAT |  |  |  |  |
| Solanaceae | *Datura discolor* | TIB | ANG |  | LOR | ANI |  |  | ALC | PAR | DAT |  |  |  |  |
| Solanaceae | *Lycium andersonii* var. *andersonii* | TIB | ANG |  |  |  |  |  |  |  |  |  |  |  |  |
| Solanaceae | *Lycium andersonii* var. *pubescens* | TIB |  | EST |  |  |  |  |  |  | DAT |  |  |  |  |
| Solanaceae | *Lycium berlandieri* var. *longistylum* | TIB |  |  | LOR |  |  |  |  |  |  |  |  |  |  |
| Solanaceae | *Lycium brevipes* var. *brevipes* | TIB | ANG | EST |  |  |  | MAR | ALC |  | DAT | SAL | RAS |  | CHO |
| Solanaceae | *Lycium californicum* subsp. *californicum* |  | ANG |  |  |  |  |  |  |  |  |  |  |  |  |
| Solanaceae | *Lycium fremontii* var. *fremontii* | TIB |  |  |  |  |  |  | ALC |  |  |  |  |  |  |
| Solanaceae | *Nicotiana clevelandii* | TIB |  |  |  |  |  |  |  |  |  |  |  |  |  |
| Solanaceae | *Nicotiana obtusifolia* | TIB | ANG | EST | LOR | ANI | NOL | MAR | ALC | PAR | DAT | SAL |  |  | CHO |
| Solanaceae | *Physalis crassifolia* var. *infundibularis* |  | ANG | EST | LOR |  |  |  |  |  |  |  |  |  |  |
| Solanaceae | *Physalis crassifolia* var. *versicolor* | TIB |  |  |  |  |  |  |  |  | DAT |  |  |  |  |
| Solanaceae | *Physalis pubescens** | TIB |  |  |  |  |  |  |  |  |  |  |  |  |  |
| Solanaceae | *Solanum hindsianum* | TIB | ANG | EST | LOR | ANI |  |  |  |  | DAT |  |  |  |  |
| Stegnospermataceae | *Stegnosperma halimifolium* | TIB | ANG | EST | LOR |  |  | MAR |  |  | DAT |  |  |  | CHO |
| Talinaceae | *Talinum paniculatum* | TIB |  |  |  |  |  |  |  |  |  |  |  |  |  |
| Tamaricaceae | *Tamarix aphylla** | TIB |  |  |  |  |  |  |  |  |  |  |  |  |  |
| Tamaricaceae | *Tamarix chinensis** | TIB | ANG |  |  |  |  |  | ALC |  |  |  |  |  |  |
| Theophrastaceae | *Bonellia macrocarpa* subsp*. pungens* | TIB |  |  |  |  |  |  |  |  |  |  |  |  |  |
| Typhaceae | *Typha domingensis* | TIB |  |  |  |  |  |  |  |  |  |  |  |  |  |
| Urticaceae | *Parietaria hespera* var. *hespera* | TIB | ANG | EST | LOR | ANI | NOL |  |  |  |  |  |  |  |  |
| Verbenaceae | *Lantana hispida* | TIB |  |  |  |  |  |  |  |  |  |  |  |  |  |
| Verbenaceae | *Lippia palmeri* | TIB |  |  |  |  |  |  |  |  | DAT |  |  |  |  |
| Violaceae | *Hybanthus fruticulosus* | TIB |  |  |  |  |  |  |  |  |  |  |  |  |  |
| Zosteraceae | *Zostera marina* var. *atam* | TIB |  |  |  |  |  |  | ALC |  |  |  |  |  |  |
| Zygophyllaceae | *Fagonia californica* | TIB |  |  |  |  |  |  |  |  | DAT |  |  |  |  |
| Zygophyllaceae | *Fagonia densa* |  | ANG |  | LOR |  |  |  |  |  |  |  |  |  |  |
| Zygophyllaceae | *Fagonia pachyacantha* | TIB | ANG |  |  |  |  |  |  |  |  |  |  |  |  |
| Zygophyllaceae | *Fagonia palmeri* | TIB |  |  |  |  |  |  |  |  |  |  |  |  |  |
| Zygophyllaceae | *Guaiacum coulteri* | TIB |  |  |  |  |  |  |  |  |  |  |  |  |  |
| Zygophyllaceae | *Kallstroemia californica* | TIB |  |  |  |  |  | MAR | ALC |  | DAT |  |  |  |  |
| Zygophyllaceae | *Kallstroemia grandiflora* | TIB |  |  |  |  |  |  |  |  |  |  |  |  |  |
| Zygophyllaceae | *Larrea* *tridentata* | TIB | ANG |  |  |  |  |  |  |  |  |  |  |  |  |
| Zygophyllaceae | *Tribulus terrestris** | TIB |  |  |  |  |  |  | ALC |  |  |  |  |  |  |
| Zygophyllaceae | *Viscainoa geniculata* var. *geniculata* | TIB | ANG | EST | LOR | ANI |  | MAR | ALC | PAR | DAT |  | RAS |  | CHO |
| **90** | **476** | **349** | **217** | **114** | **85** | **45** | **58** | **29** | **54** | **18** | **101** | **18** | **14** | **14** | **31** |
