# Supplementary material for: Controls of plant diversity and composition on a desert archipelago
Source: PeerJ. 2019 Jul 9;7:e7286. doi: 10.7717/peerj.7286 (PMC6625499; doi:10.7717/peerj.7286)
Supplement: Supplemental Information 2 [file peerj-07-7286-s002.docx]

SUPPORTING INFORMATION

Controls of plant diversity and composition on a desert archipelago

Benjamin T. Wilder, Richard S. Felger, Exequiel Ezcurra

**Supplemental Appendix S2. Alternative derivation of the species-area curve**

The species-area curve belongs within a larger group of species-accumulation functions (Soberón and Llorente 1993). It can be derived from the differential equation

*dS*/*dn* = *z* (*S*/*n*) (1)

which basically states that, given a collection of *n* specimens, the probability of finding a new species when an individual is randomly collected is proportional to the number of species that have already been collected (*S*). Alternatively, given a collection of *S* species, the probability of finding a new species when an individual is randomly collected is inversely proportional to the completeness of the collection effort, or the number of individuals that have already been collected (*n*). That is, the more species we have collected with a certain fixed collection effort, the more likely that there may be species in the field yet to be collected, and, alternatively, the more complete the collection vouchering a given number of species, the less likely that there may be unseen species left in the field.

Integrating the above equation, we get log *S* = *C* + *z* log *n*, where *C* is an integration constant, or, alternatively

*S* = *c n^z^* (2)

where the constant *c* is equal to exp(*C*). Now, if we assume that the average area occupied by each individual is more or less equal in all islands, then it follows that

*A* = *ρ n* (3)

where *A* is the island’s area and *ρ* is the area occupied, on average, by each individual. Substituting *n* for *A* in eq. 2 we get the familiar species-area power function:

*S* = *k A^z^* (4)

where the constant *k* = c *ρ ^z^* is the number of species found within a unit area. Thus, Preston’s canonical model is not really necessary to deduce the species-area power function. Its main importance lies in the fact that it provides a yardstick for comparing the power exponent values. Indeed, if species abundances in the source community follow a lognormal distribution, then it is expected that the exponent *z* in random samples of different sizes should show a value of ca. 0.263.
